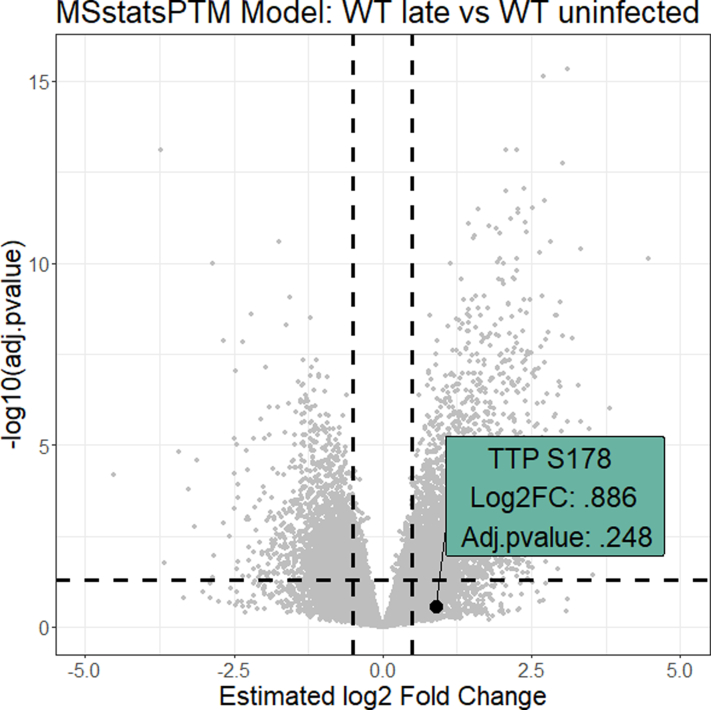# MSstatsPTM: Statistical Relative Quantification of Posttranslational Modifications in Bottom-Up Mass Spectrometry-Based Proteomics

**DOI:** 10.1016/j.mcpro.2022.100477

**Published:** 2022-12-08

**Authors:** Devon Kohler, Tsung-Heng Tsai, Erik Verschueren, Ting Huang, Trent Hinkle, Lilian Phu, Meena Choi, Olga Vitek

**Affiliations:** 1 Khoury College of Computer Sciences, Northeastern University, Boston, Massachusetts, USA; 2 Department of Mathematical Sciences, Kent State University, Kent, Ohio, USA; 3 ULUA BV, Antwerp, Belgium; 4 MPL, Genentech, South San Francisco, California, USA

**Keywords:** mass spectrometry, statistics, posttranslational modifications, quantification, bioinformatics software, ANOVA, Analysis of variance, DDA, Data-dependent acquisitions, DIA, Data-independent acquisition, FDR, false discovery rate, GSDMD, Gasdermin D, KGG, Ub-remnant diglycyl-lysine, MS, mass spectrometry, PTM, Posttranslational modifications, PPV, positive predictive value, SRM, Selected reaction monitoring, TMT, Tandem mass tag

## Abstract

Liquid chromatography coupled with bottom-up mass spectrometry (LC-MS/MS)–based proteomics is increasingly used to detect changes in posttranslational modifications (PTMs) in samples from different conditions. Analysis of data from such experiments faces numerous statistical challenges. These include the low abundance of modified proteoforms, the small number of observed peptides that span modification sites, and confounding between changes in the abundance of PTM and the overall changes in the protein abundance. Therefore, statistical approaches for detecting differential PTM abundance must integrate all the available information pertaining to a PTM site and consider all the relevant sources of confounding and variation. In this manuscript, we propose such a statistical framework, which is versatile, accurate, and leads to reproducible results. The framework requires an experimental design, which quantifies, for each sample, both peptides with PTMs and peptides from the same proteins with no modification sites. The proposed framework supports both label-free and tandem mass tag-based LC-MS/MS acquisitions. The statistical methodology separately summarizes the abundances of peptides with and without the modification sites, by fitting separate linear mixed effects models appropriate for the experimental design. Next, model-based inferences regarding the PTM and the protein-level abundances are combined to account for the confounding between these two sources. Evaluations on computer simulations, a spike-in experiment with known ground truth, and three biological experiments with different organisms, modification types, and data acquisition types demonstrate the improved fold change estimation and detection of differential PTM abundance, as compared to currently used approaches. The proposed framework is implemented in the free and open-source R/Bioconductor package MSstatsPTM.

## Highlights

• MSstatsPTM differentiates statistical changes in PTMs and global protein abundances.
• Novel methodology removes confounding between the modification and global protein.
• MSstatsPTM is an open-source R package available on Bioconductor.
• The package is applicable to a variety of experimental designs and labeling methods.
• Benchmarked on simulated data, a spike-in mixture, and biological investigations.

## Graphical Abstract

**Figure:**
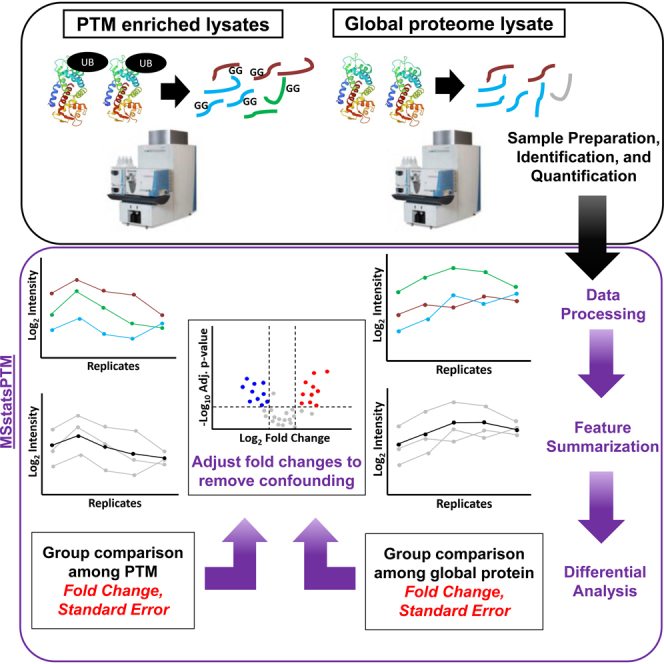

## In Brief

MSstatsPTM detects statistical differences between posttranslational modifications and global protein abundances. It includes novel methodology to remove confounding between the effect of individual modification *versus* the global protein. MSstatsPTM is implemented as an open-source R package available on Bioconductor. The package can be applied to a variety of experimental designs, including those which are label-free and label-based. The package has been benchmarked on several datasets including simulated datasets, a spike-in controlled investigation, and multiple biological experiments.

Signaling mechanisms allow cells to mount a fast and dynamic response to a multitude of biomolecular events. Signaling is facilitated by the modification of proteins at specific residues, acting as molecular on/off switches (1, 2, 3). Characterizing relative abundance of a modification site’s occupancy repertoire across experimental conditions provides important insights (4). For example, meaningful patterns of changes in posttranslational modifications (PTMs) abundance can serve as biomarkers of a disease (5). Alternatively, distinguishing the quantitative changes in a PTM from the overall changes of the protein abundance helps gain insight into biological and physiological processes operating on a very short timescale (6, 7, 8). This helps to distinguish between relative site occupancy changes at steady-state protein levels, typical for short timescale signaling events, and observed relative changes of PTMs as a result of underlying gene expression or protein abundance levels.

Bottom-up liquid chromatography coupled with tandem mass spectrometry (LC-MS/MS) is a tool of choice for unbiased and large-scale identification and quantification of proteins and their PTMs (9, 10). However, LC-MS-based interrogation of the modified proteome is challenging, for a number of reasons. First, the relatively lower abundance of modified proteoforms dictates that a global interrogation can only be achieved through large-scale enrichment protocols with modification-specific antibodies or beads (11). Variability in the enrichment efficiency inevitably affects the reproducibility of the number of spectral features (*e.g.*, peptide precursor ions or their fragments) and their intensities. Second, contrary to the often large number of identified peptides that can be used to quantify protein abundance, there are relatively few representative peptides that span a modification site, and there may be multiple modified sites on a single peptide (28). Third, unless early signaling events are interrogated, the interpretation of the relative changes in modification occupancy are inherently confounded with changes in the overall protein abundance, complicating the interpretation of the results (6, 12). Finally, technological aspects of bottom-up MS experiments, such as presence of labeling by tandem mass tag (TMT), introduce additional sources of uncertainty and variation.

The technological difficulties in PTM identification and quantification increase the uncertainty and the variation in the data and challenge the downstream statistical analyses. Frequently data from these experiments are analyzed using statistical methods that were not originally designed for this task. Researchers use methods such as *t* test (13), analysis of variance (ANOVA) (14), or Limma (15), by taking as input the intensity ratios of modified and unmodified peptide summaries and comparing the mean abundance of different PTM sites. Such approaches do not fully account for all the sources of uncertainty. As the result, these approaches are either not directly applicable to experiments with nontrivial designs (such as experiments with multiple conditions, paired and time course designs, and experiments with labeling) or require the analysts to exercise nontrivial statistical expertise.

This manuscript proposes a versatile statistical analysis framework that accurately detects relative changes in PTMs. The framework requires an experimental design, which quantifies, for each sample, both the peptides with PTMs and peptides from the same proteins with no modification sites. The framework supports data-dependent acquisitions (DDAs) that are label-free or TMT-based. The statistical methodology separately summarizes the abundances of peptides with and without the modification sites and fits separate linear mixed effects models that reflect the biological and technological aspects of the experimental design. Unmodified peptides may or may not span a modifiable site. Next, model-based inferences regarding the PTM and the unmodified protein-level summaries are combined to account for the confounding between these two sources.

We evaluated the proposed framework on two datasets from computer simulations, one benchmark controlled mixture and three biological investigations. The datasets illustrate a diverse set of organisms, modification types, acquisition methods, and experimental designs, showing the applicability of the framework to a variety of situations. By appropriately leveraging the information from the unmodified portion of the protein sequence, the proposed approach improved the accuracy of the estimates of PTM fold changes and produced a better calibrated false positive (FP) rate of detecting differentially abundant PTMs as compared to existing methods. In particular, accounting for the confounding from unmodified protein abundance allowed us to characterize the true effect of the modification, avoiding the need for more manual and time intensive follow-up investigation.

The proposed approach is implemented as a freely available open source R package MSstatsPTM, as part of the MSstats family of packages (16, 17), and is available on Bioconductor.

## Experimental Procedures

### Data Overview and Availability

Table 1 summarizes the experiments. Two computer simulations had known ground truth and varied in experimental realism. The first simulation produced a perfectly clean dataset, with many replicates and no missing values. The second simulation introduced real-world characteristics, such as limited modified features and missing values. Details of computer simulations are available in Supplementary Sec. 3.1 & 3.2, and on GitHub (https://github.com/devonjkohler/MSstatsPTM_simulations).

**Table 1:** Simulated and experimental datasets

Experimental type	Dataset	No. of conditions	No. of bio. replicates	No. of mod. peptides	No. of mod. features/site	No. of unmod. Features/site	Data availability	Analysis
Known ground truth	Computer Simulation 1—Label-free	2/3/4	2/3/5/10	1000	10	10	Github	
	Computer Simulation 2—Missing and low features	2/3/4	2/3/5/10	1000	2	10	Github	
	Spike-in benchmark—Ubiquitination—Label-free	4	2	12,137	1.37	10.17	MSV000088971	RMSV000000669
Biological experiment	Human—Ubiquitinatio=n—1mix-TMT	6	2 or 1	8848	1.21	11.01	MSV000088966	RMSV000000356
	Mouse—Phosphorylation—2mix-TMT	6	4 or 3	26,433	1.67	11.61	MSV000085565	RMSV000000357
	Human—Ubiquitination—Label-free	6	2	10,799	1.40	1.65	MSV000078977	RMSV000000358

“Dataset” is the dataset code name. “No. of bio. replicates” shows the number of biological replicates per condition. Simulations were generated with different numbers of replicates. The designs of two biological experiments were unbalanced with unequal replicates per condition. “No. of mod. features/site” is the number of features (*i.e.*, peptide ions) used to estimate the abundance of a single modification. “No. of unmod. peptides/protein” is the number of peptide ions without modifications that were used to estimate the global protein abundance. “Data availability” is the ID of the MassIVE.quant repository or the GitHub repository. “Analysis” is the ID of the MassIVE.quant reanalysis container, containing analysis code and modeling results. All the experiments were conducted in data-dependent acquisition (DDA) mode.

One spike-in experiment also had known changes in modified spike-in peptides but had real world experimental characteristics. Finally, three biological experiments demonstrated the applicability of the proposed approach across different biological organisms, modifications, experimental designs, and acquisition strategies. All biological experiments used a modified version of the AScore algorithm (cutoff=15) for site localization (18). The experimental data, R scripts with MSstatsPTM analysis, and results of the statistical analysis are available in MassIVE.quant (https://massive.ucsd.edu/ProteoSAFe/static/massive-quant.jsp) (19).

### Dataset 1: Computer Simulation 1—Label-Free Clean

#### Simulation Design

The simulation represented an idealistic case. Twenty-four synthetic label-free datasets were generated with different experimental designs and different biological variation. In each dataset, 1000 proteins had 10 unmodified features per protein. Each of the 1000 proteins had one PTM. Each PTM was represented by 10 modified features. The PTMs of 500 proteins had a differential fold change between conditions, while the other 500 proteins were generated with no changes in abundance between conditions. Furthermore, the fold changes of half of the 500 differential PTMs were fully masked by changes in the unmodified portion of the protein. Finally, the fold change of half the 500 nondifferential PTMs was entirely due to changes in the unmodified portion of the protein. All the differential PTMs were generated with an expected log base two fold change of 0.75 between conditions.

Each simulation was generated with random biological variation. The observed peptide abundances were simulated by adding random noise $N\left(0,\sigma^{2}\right)$ to the deterministic abundances described above. Two values $\sigma^{2}=\left\{.2,.3\right\}$ were motivated by the experimental datasets in this manuscript.

#### Evaluation

We evaluated the ability of the statistical methods to correctly detect differentially abundant PTMs. We gauged the ability of the methods to avoid FPs (*i.e.*, specificity), accurately estimate the fold change between conditions, and analyzed the sensitivity of detecting differentially abundant PTMs. The evaluation was performed both in the presence of confounding with changes in the unmodified protein and after applying adjustment to correct for the confounding.

### Dataset 2: Computer Simulation 2—Label-Free With Few Low Feature Counts and Missing Values

#### Simulation Design

The data were simulated as above, while providing a more realistic representation of the experiments. The feature counts and the proportion of missing values were as observed on average over all the experimental datasets in this manuscript. Specifically, PTMs were simulated with two modified peptide features, and unmodified portions of the protein were simulated with 10 features. Additionally, 20% of observations for both modified and unmodified peptides were missing completely at random.

#### Evaluation

The methods were evaluated as above. We evaluated their ability to correctly detect PTM’s specificity, fold change estimation, and sensitivity. These statistics were analyzed both in the presence of and without confounding with the overall changes in protein abundance.

### Dataset 3: Spike-In Benchmark—Ubiquitination—Label-Free

#### Experimental Design

**Fig. 1:**
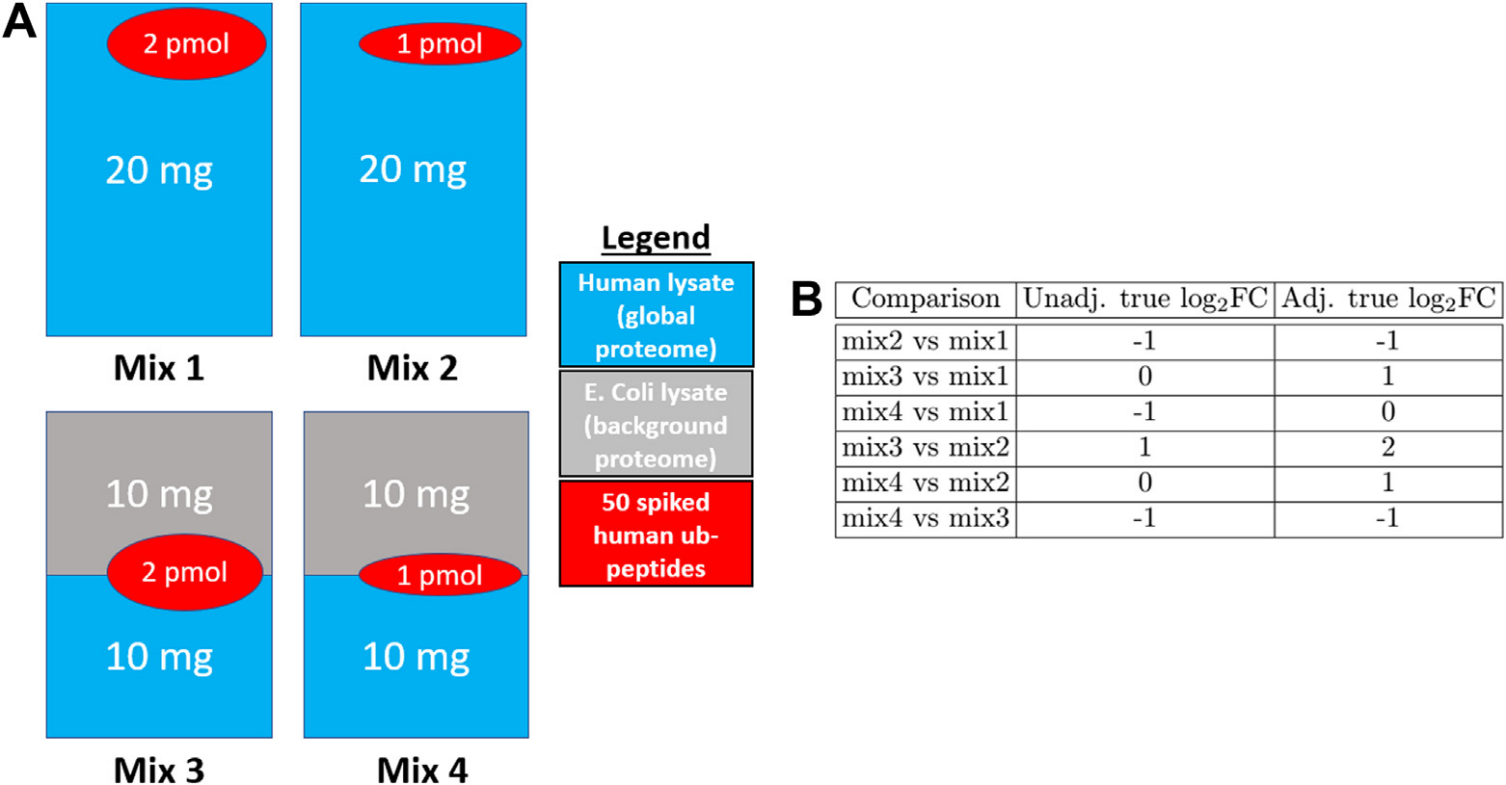
**Dataset 3: Spike-in benchmark—ubiquitination—label-free.***A,* four mixtures (*i.e.*, conditions) were created with varying amounts of human lysate, background *E. coli* lysate, and human spike-in Ub-peptide mixture. Unmodified peptides from human lysate were viewed as the global proteome. Background *E. coli* lysate were used to equalize total protein levels. Fifty heavy-labeled KGG motif peptides from 20 human proteins were spiked into the mixed background of the lysates. Quantitative changes in protein and site abundance of these 20 human proteins were the target of the benchmark. *B,* we distinguished the unadjusted changes (*i.e.*, changes in the abundances of the modified peptides) and the protein-level adjusted changes of (*i.e.*, changes in the abundances of the modified peptides relative to the changes in the abundances of the human lysate). “Unadj. true log_2_FC” are the log-ratios of the abundances of the spiked peptides between each condition. “Adj. true log_2_FC” was calculated by determining the ratios of the abundances of the spiked peptides and human lysate between each condition and then adjusting the ratio of the spiked peptides by the human lysate, similarly to (Equation 2).

Fig. 1*A* overviews the experimental design. Four mixtures (*i.e.*, conditions) were created with varying amounts of human lysate, background *E. coli* lysates, and human spike-in Ub-peptide mixture. Unmodified peptides from human lysate were viewed as the global proteome. Background *E. coli* lysate were used to equalize total protein levels. Fifty heavy-labeled Ub-remnant diglycyl-lysine (KGG) motif peptides from 20 human proteins were spiked into the mixed background of the lysates. Quantitative changes in protein and site abundance of these 20 human proteins were the target of the benchmark. In particular, we distinguished the unadjusted changes (*i.e.*, changes in the abundances of the modified peptides) and the protein-level adjusted changes of (*i.e.*, changes in the abundances of the modified peptides relative to the changes in the abundances of the human lysate). The true log-fold changes between the relevant components of the relevant mixtures are summarized in Fig. 1*B*. Two replicate mixtures were created per condition.

#### Data Acquisition

A detailed overview of the methods used to acquire this data is available in Supplementary Sec. 1. Each mixture was analyzed with KGG enrichment and without KGG enrichment (*i.e.*, in a global profiling run), with label-free LC-MS/MS. There was a 90.2% overlap of protein identifications between the identified background-modified peptides and proteins quantified in the global profiling run.

#### Evaluation

We expect the relative abundances of the spike-in peptides to change as in Fig. 1*B*. The changes in peptide abundances in all the comparisons except Mix 4 *versus* Mix 1 were distinct from changes in the global proteome abundances and distinct from zero and were viewed as positive controls. In the comparison of Mix4 *versus* Mix 1, both the modified peptides and the global proteome background changed two-fold, and as the result, the peptides in this comparison were viewed as a negative control. The background *E. Coli* lysate peptides were not expected to change in abundance in comparison, after accounting for adjustment, and were viewed as additional negative controls. We evaluated the ability of the statistical methods to avoid FPs, as well as their sensitivity in detecting the differentially abundant spike-in peptides and accurately estimate their expected fold change.

### Dataset 4: Human—Ubiquitination—1mix-TMT

#### Experimental Design

Luchetti *et al*. (20) profiled human epithelial cells engineered to express IpaH7.8 under a dox inducible promoter. Uninfected cells were measured at 0 and 6 h, while cells infected with *Shigella flexneri* (*S. flexneri*) bacteria were measured at 1, 2, 4, and 6 h increments, resulting in six total conditions. 11 samples were allocated to one TMT mixture in an unbalanced repeated measure design. All conditions had two biological replicates except for the Dox1hr condition, which was allocated one replicate.

#### Data Acquisition

The search parameters and data acquisition were as described in (20). The ubiquitinated peptides and the total proteome (*i.e.*, global profiling) were each conducted in a single LC-MS/MS run. There was a 95% overlap between the identified modified peptides and proteins that were quantified in the global profiling run.

#### Evaluation

We evaluated the ability of the statistical methods to detect changes in the abundance of modified peptides both before and after adjusting for changes in global protein abundance. The six conditions were labeled Dox1hr, Dox2hr, Dox4hr, Dox6hr, NoDox0hr, and NoDox6hr. All conditions were compared with each other, resulting in 15 pairwise comparisons. Since the dataset was a biological investigation, the true positive modifications were unknown. *Shigella* ubiquitin ligase IpaH7.8 was shown to function as an inhibitor of the protein Gasdermin D (GSDMD). GSDMD was actively degraded when IpaH7.8 expression was induced by dox treatment in human cells. We expect IpaH7.8 to function as an inhibitor of GSDMD in the global profiling run.

### Dataset 5: Mouse—Phosphorylation—2mix-TMT

#### Experimental Design

Maculins *et al*. (21) studied primary murine macrophages infected with *S. flexneri*. The experiment quantified the abundance of total protein and of phosphorylation in wildtype (WT) and in ATG16L1-deficient (cKO) samples, uninfected and infected with *S. flexneri*. The abundance of total protein and PTMs were quantified at three time points, uninfected, early infection (45–60 min), and late infection (3–3.5 h). Twenty-two biological samples were allocated to two TMT mixtures in an unbalanced repeated measure design, with 11 samples allocated to each mixture. 16 replicates were spread equally between the early and late WT and cKO conditions, resulting in four replicates per condition. Both the uninfected WT and cKO contained three replicates, with mixture one allocating one replicate to uninfected WT and two replicates to uninfected cKO. Conversely, mixture two contained one replicate of uninfected cKO and two uninfected WT.

#### Data acquisition

The search parameters and data acquisition were as described in (21). This experiment included a total proteome (*i.e.*, a global profiling run) and a phosphopeptide enrichment run. There was a 90% overlap between the identified modified peptides and proteins that were quantified in the global profiling run.

#### Evaluation

We evaluated the ability of the statistical methods to detect changes in the abundance of modified peptides both before and after adjusting for changes in global protein abundance. The six conditions were labeled KO Uninfect, KO Early, KO Late, WT Uninfect, WT Early, and WT Late. Nine total comparisons were made, namely KO Early-WT Early, KO Late-WT Late, KO Uninfected-WT Uninfected, KO Early-KO Uninfected, KO Late-KO Uninfected, WT Early-WT Uninfected, WT Late-WT Uninfected, Infected-Uninfected, and KO-WT. Since the dataset was a biological investigation, the true positive modifications were unknown.

### Dataset 6: Human—Ubiquitination—Label-Free No Global Profiling Run

#### Experimental Design

Cunningham *et al*. (22) investigated the relationship between USP30 and protein kinase PINK1 and their association with Parkinson’s Disease. The experiment profiled ubiquitination sites and analyzed changes in the modified site abundance. The experiment had four conditions, CCCP, USP30 over expression (USP30 OE), Combo, and Control. Cell lines were used to create two biological replicates per condition. The abundance of modified peptides was quantified with label-free LC-MS/MS.

#### Data Acquisition

The search parameters and data acquisition were as described in (22). This experiment did not include a separate global profiling run to quantify unmodified peptides. In addition to low feature counts for unmodified peptides, this led to substantially fewer matches between modified and unmodified peptides. There was a 41.9% overlap between the identified background modified peptides and proteins that were quantified in the global profiling run.

#### Evaluation

We evaluated the ability of the statistical methods to detect changes in the abundance of modified peptides both before and after adjusting for changes in global protein abundance. All the conditions were compared with each other in a full pairwise comparison, resulting in six comparisons. Since the dataset is a biological investigation, the true positive modifications were unknown.

### Background

#### Goals of PTM Characterization, Input to Statistical Analyses, and Notation

**Fig. 2:**
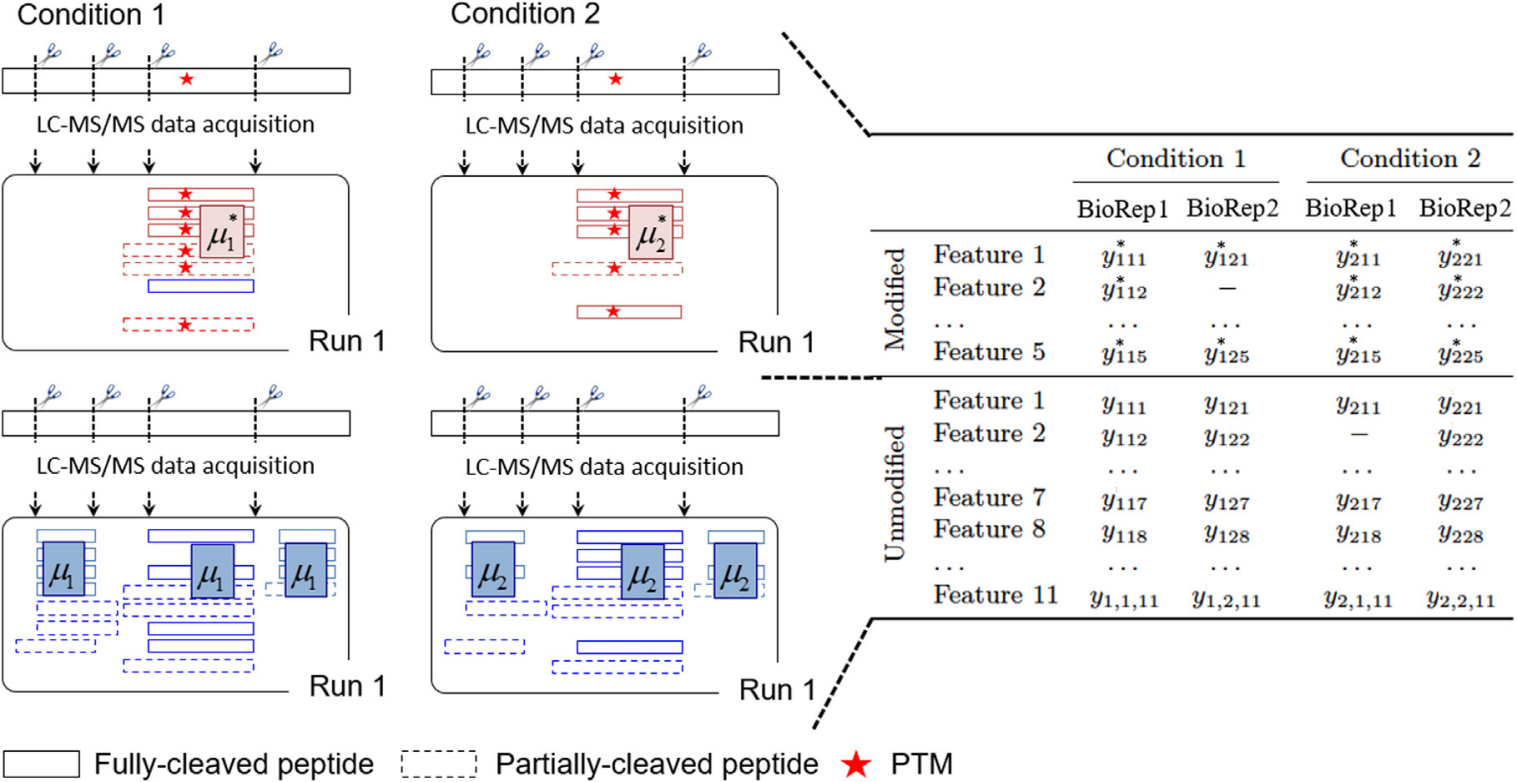
**Schematic representation of one PTM site, in a special case of a label-free experiment with**I=2**conditions and**J=2**biological replicates per condition.** After a log_2_ transform, we are interested in estimating the difference between the population-level PTM abundance between condition 1 and condition 2 (*i.e.*, $\mu_{1}^{*}-\mu_{2}^{*}$ ), relative to the population-level difference of the overall protein abundance (*i.e.*, $\mu_{1}-\mu_{2}$). These quantities are characterized by the observed spectral features (*boxes*), *i.e.*, peptides of different charge states. The peptides can be fully cleaved (*solid lines*) or partially cleaved (*dashed lines*). Unmodified features (*blue*) in the enriched runs are removed. The log_2_ intensities of the modified peptides in condition i, run j, and feature k are denoted by $y_{ijk}^{*}$. The log_2_ intensities of feature l corresponding to the unmodified peptide in condition i and run j are denoted by $y_{ijl}$. PTM, posttranslational modification.

Consider a label-free LC-MS/MS experiment in the special case of a balanced design with I conditions and J biological replicates per condition. For simplicity, we assume that the experiment has no technical replicates, such that each biological replicate is represented by a single LC-MS/MS run. Fig. 2 schematically illustrates this data structure for one protein and one PTM site, I=2 and J=2. For one protein, the PTM site is represented by K spectral features (*i.e.*, peptide ions, distinguished by their cleavage residues and charge states). The number of modified and unmodified features typically varies across proteins. Some log_2_ intensities can be outliers, and some spectral features can be missing. The log_2_ intensity of Feature k, in Replicate j of Condition i is denoted by $y_{ijk}^{*}$. Conversely, the unmodified portion of the protein is represented by L spectral features, and the log_2_ intensity of Feature l from the unmodified portion of the protein in the same run is denoted by $y_{ijl}$. The features can be quantified as part of a same mass spectrometry run or in a separate enrichment and global proteome profiling run.

The population quantity of interest is the difference between the log_2_ abundances of a PTM site in Condition i and Condition i′, denoted by $\mu_{i}^{*}$ and $\mu_{i\prime }^{*}$ respectively. We are interested in testing the null hypothesis

(1) $$H_{0}:\Delta_{PTM}=\mu_{i}^{*}-\mu_{i\prime }^{*}=0vsH_{a}:\Delta_{PTM}=\mu_{i}^{*}-\mu_{i\prime }^{*}\neq 0$$

Unfortunately, this population quantity is inherently confounded with the overall changes in protein abundance. To account for this, it is advantageous to consider a different null hypothesis:

(2) $$H_{0}:\Delta_{adj}=\left(\mu_{i}^{*}-\mu_{i}\right)-\left(\mu_{i\prime }^{*}-\mu_{i\prime }\right)=0vsH_{a}:\Delta_{adj}=\left(\mu_{i}^{*}-\mu_{i}\right)-\left(\mu_{i\prime }^{*}-\mu_{i\prime }\right)\neq 0$$

where $\mu_{i}$ and $\mu_{i\prime }$ reflect the overall log_2_ protein abundances in Condition i and Condition i′. These quantities are estimated using protein features with and without the modification site.

### Existing Statistical Methods for Detecting Differentially Abundant PTMs

#### ANOVA on Summarized Modified Log_2_ Intensities

ANOVA (23) is the simplest statistical model for summarized modified features in each biological replicate. The summarization often consists of averaging (or taking the median or other robust summary) of the log_2_ intensities of the modified features in each replicate, *e.g.,* ${\hat{y}}_{ij}^{*}=\sum_{k=1}^{K}y_{ijk}^{*}/K$. Alternatively, summarization sums the intensities of the modified features on the original scale and then takes the log_2_

(3) $$\begin{array}{l} {\hat{y}}_{ij}^{*}=\log_{2}\left(\sum_{k=1}^{K}2^{y_{ijk}^{*}}\right) \end{array}$$

The basic ANOVA model is then

(4) $$\begin{array}{l} {\hat{y}}_{ij}^{*}=\mu_{i}^{*}+\varepsilon_{ij}^{*},\varepsilon_{ij}^{*}\overset{iid}{∼}N\left(0,\sigma^{*2}\right),i=1,\ldots ,I,j=1,\ldots ,J \end{array}$$

The model allows us to estimate ${\hat{\Delta }}_{PTM}$ and its standard error. The estimates are used to test the null hypothesis in (Equation 1) by comparing the model-based test statistic against the Student distribution with df=I(J−1) degrees of freedom in balanced designs. Unfortunately, this approach is fundamentally flawed as it does not account for the confounding between changes in the PTM abundance and the overall changes in the abundance of the unmodified portion of the protein.

### ANOVA Based On Ratios of Modified and Unmodified Log_2_ Intensities

The basic ANOVA can be extended to account for the confounding of changes in PTM abundance and overall changes in protein abundance (24, 25, 26). Typically this is done by first calculating sums of the intensities of the modified and unmodified features on the original scale and then considering replicate-wise ratios of the sums and taking the log_2_

(5) $$\begin{array}{l} u_{ij}=\log_{2}\frac{\sum_{k=1}^{K}2^{y_{ijk}^{*}}}{\sum_{l=1}^{L}2^{y_{ijl}}}=\log_{2}\left(\sum_{k=1}^{K}2^{y_{ijk}^{*}}\right)-\log_{2}\left(\sum_{l=1}^{L}2^{y_{ijl}}\right) \end{array}$$

The approach then models these values with the basic ANOVA, which corresponds to

(6) $$u_{ij}=\left(\mu_{i}^{*}-\mu_{i}\right)+{\varepsilon \prime }_{ij},where\;{\varepsilon \prime }_{ij}\overset{iid}{∼}N\left(0,{\sigma \prime }^{2}\right),i=1,\ldots ,I,j=1,\ldots ,J$$

The model allows us to estimate ${\hat{\Delta }}_{adj}$ and its standard error. Based on this model, we can test the more relevant null hypothesis in (Equation 2), by comparing the test statistic against the Student distribution with df=I(J−1) degrees of freedom in balanced designs.

Although effective, the approach is somewhat simplistic. It is not applicable to experimental designs with more complex sources of biological and technological variation, such as experiments with repeated measurements, experiments with multiple batches, or experiments with TMT labeling. Since (Equation 5) performs the adjustment on the replicate level, the experiment must contain a matching number of replicates in both the modified and unmodified runs. Technological artifacts such as missing values further undermine the calculation of $u_{ij}$ in (Equation 5). Finally, there is no self contained, straightforward implementation of the method, such as in the form of a coding package, and therefore the approach requires a manual implementation.

### Limma

The estimation of nuisance variation of the two ANOVA models above is often further expanded with Empirical Bayes moderation implemented in Limma (15, 24, 27, 28, 29, 30). A typical application of Limma on summarized modified log_2_ intensities takes as input ${\hat{y}}_{ij}^{*}$, obtained as in (Equation. 3), and for each PTM fits the linear model in (Equation 4). A typical application of ratio based Limma takes as input $u_{ij}$, obtained as in (Equation 5), and for each PTM fits the linear model in (Equation 6). The Limma versions of the models differ from the models in (Equations 4 and 6) in that they specify additional prior distributions for the model parameters. The priors are estimated from the same data by combining the information across all the proteins and all the PTM as described in (27). Testing the null hypothesis is enhanced by combining the PTM and protein-specific estimates of variation with a consensus estimate obtained from the estimated priors. As the result, in experiments with few biological replicates the standard errors are often smaller, and the degrees of freedom are often larger than without moderation (15). Thus, the approach tends to increase the sensitivity of detecting differential abundance.

Since Limma only improves upon the estimation of variation, its limitations are similar to those of ANOVA. In particular, the method is only directly applicable to experiments with at most two variance components and cannot account for all the sources of variation in experiments with either isobaric labeling or complex designs. There is no self contained implementation of the methods to PTMs, requiring manual transformation and application by the user.

### Isobar-PTM

Isobar-PTM was also proposed for experiments with LC-MS/MS quantitative strategies that employ isobaric labels such as TMT or isobaric tag for relative and absolute quantification (31). Isobar-PTM expresses MS measurements with a linear model and performs adjustment with respect to protein abundance using the difference between log-ratio of modified peptides in two channels and log-ratio of protein level. Unfortunately, this statistical modeling framework is not applicable to either label-free workflows or experiments with complex designs.

### Relative Protein Quantification in MSstats

**Supplemental Figure 1:**
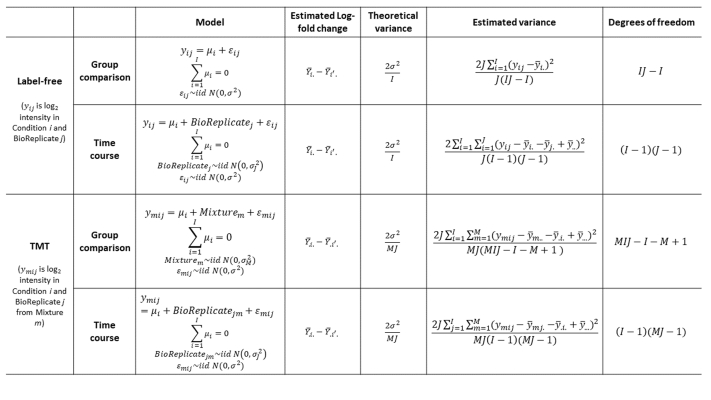


MSstats (16) and MSstatsTMT (17) are a family of R/Bioconductor packages for statistical relative quantification of proteins and peptides in global, targeted, and data-independent proteomics. The packages take as input log_2_ intensities $y_{ijk}$. For each protein, the log_2_ intensities are first summarized into a single value per protein per run ${\hat{y}}_{ij}$ using Tukey’s median polish (32). The summaries are then used as input to fit a flexible family of linear mixed-effects models (33, 34, 35). The models are fit separately for each protein. The specific model depends on the design of the experiment, labeling type, and data acquisition type as summarized in supplemental Fig.S1. For example, the unmodified protein features in the simple design in Fig. 2 are modeled with one-way ANOVA

(7) $$\begin{array}{l} {\hat{y}}_{ij}=\mu_{i}+\varepsilon_{ij},where\varepsilon_{ij}\overset{iid}{∼}N\left(0,\sigma^{2}\right) \end{array}$$

In contrast, a group comparison experiment with multiple TMT mixtures is modeled as

(8) $$\begin{array}{l} {\hat{y}}_{imj}=\mu_{i}+Mixture_{m}+\varepsilon_{imj},where\;Mixture_{m}\overset{iid}{∼}N\left(0,\sigma_{M}^{2}\right),\varepsilon_{imj}\overset{iid}{∼}N\left(0,\sigma^{2}\right) \end{array}$$

Moreover, the model fit for a particular protein depends on the pattern of missing values in that protein. If some of the terms of the model reflecting the experimental design are not estimable, a simpler model is fit for that protein instead.

Parameters of the model are estimated using restricted maximum likelihood (36). The parameters allow us to estimate the pairwise comparison ${\hat{\Delta }}_{protein}={\hat{\mu }}_{i}-{\hat{\mu }}_{i\prime }$ and its standard error. Similarly to Limma, MSstatsTMT includes an optional Empirical Bayes moderation of the standard error (17), increasing the sensitivity of detecting differential abundance when the number of biological replicates in each condition is small.

MSstats and MSstatsTMT can also be used at the feature or at the modification site level, as opposed to protein level. For example, summarizing the features per PTM site instead of per protein, the approach allows us to test the null hypothesis in (Equation 1).

The MSstats framework has a number of advantages over the methods above. First, unlike ANOVA and Limma, MSstats and MSstatsTMT are applicable to arbitrary complex experimental designs, including designs with multiple sources of variation and unbalanced designs. Second, the approach is applicable to various data acquisition types, including label-free DDA and data-independent acquisition (DIA) and experiments with TMT labeling. Third, the MSstats packages are compatible with various data processing tools such as Skyline, Spectronaut, MaxQuant, Progenesis, Proteome Discoverer, and OpenMS. Finally, the custom MSstats and MSstatsTMT implementation accounts for potential data artifacts is numerically scalable and stable and is available through both command line and a dedicated graphical user interface.

Unfortunately, the MSstats framework focuses on overall protein abundance and as the result tests the null hypothesis in (Equation 1). It does not account for the confounding between the changes in PTM abundance and the overall changes in protein abundance. This manuscript proposes a simple extension to the methodology in MSstats and MSstatsTMT to test the null hypothesis in (Equation 2).

## Results

### Statistical Methods in MSstatsPTM

#### Detecting Changes in PTMs, Adjusted for Global Changes in Protein Abundance

**Fig. 3:**
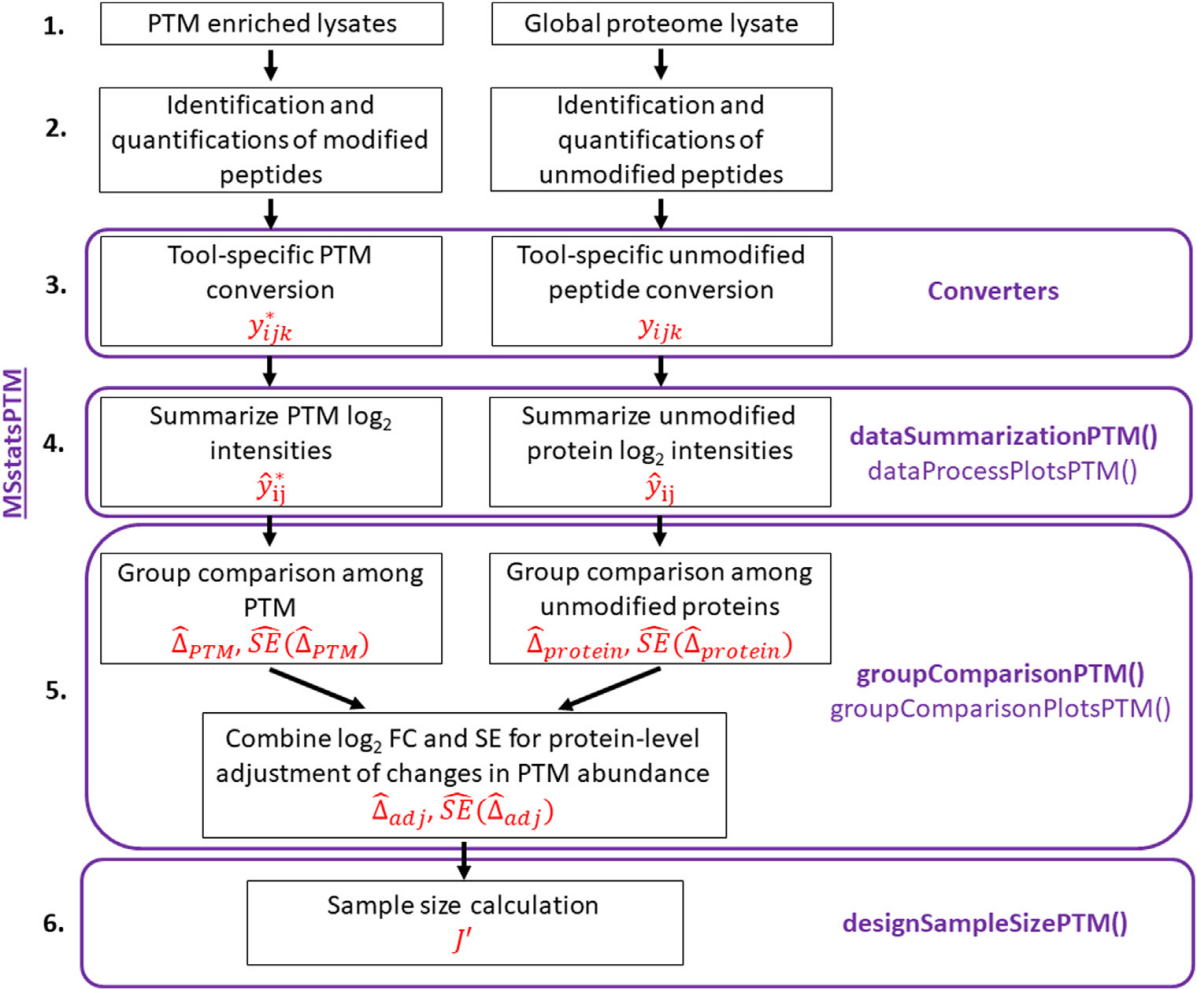
**The MSstatsPTM workflow**. The names of the MSstatsPTM R functions used for each step are highlighted in *purple* and the output notations are highlighted in *red*. The workflow begins with the acquisition of the enriched and global proteome lysates. The package is applicable to label-free data acquisitions, such as DDA, DIA, SRM, and label-based data acquisitions, such as TMT. It takes as input lists of identified and quantified spectral features for the PTM and for the unmodified portion of the protein, produced by spectral processing tools such as MaxQuant, Progenesis, or Spectronaut. Conversion, summarization and statistical modeling are performed separately for the PTM and for the unmodified portions of the proteins. Steps 4 and 5 leverage the summarization and modeling functions from MSstats and MSstatsTMT. Model-based summaries are combined to adjust the changes in the PTM abundance for changes in abundance of the unmodified portion of the protein. Finally, sample size calculation for future experiments can be performed using the modeling output. DDA, data-dependent acquisitions; DIA, data-independent acquisition; PTM, posttranslational modification; SRM, selected reaction monitoring.

The overall statistical analysis workflow and its implementation are summarized in Fig. 3. MSstatsPTM takes as input the modified spectral features $y_{ijk}^{*}$ and the corresponding unmodified features $y_{ijk}$. Ideally, the modified features are acquired separately after an enrichment to maximize the information content in the resulting dataset, and the unmodified features are acquired separately as part of a global proteome profiling. However, the method can also take as input a combination of modified and unmodified features acquired within a same run.

Each feature type is first analyzed separately, with MSstatsPTM methods calling the relevant functionalities in MSstats (for label-free experiments) or MSstatsTMT (for experiments with TMT labels). In particular, the modified features are summarized into run-level summaries ${\hat{y}}_{ij}^{*}$.

The estimated summaries of the modified features are used as the input to models such as in (Equation 7) or (Equation 8). The resulting model-based estimates include ${\hat{\Delta }}_{PTM}={\hat{\mu }}_{i}^{*}-{\hat{\mu }}_{i\prime }^{*}$ and its standard error $\hat{SE}\left({\hat{\Delta }}_{PTM}\right)$. Similarly, the unmodified features of each protein are summarized for each run into ${\hat{y}}_{ij}$, and the summaries are used as input to a separate analysis by MSstats or MSstatsTMT producing ${\hat{\Delta }}_{protein}={\hat{\mu }}_{i}-{\hat{\mu }}_{i^{\prime }}$ and $\hat{SE}\left({\hat{\Delta }}_{protein}\right)$. From these summaries, the proposed approach estimates the adjusted difference ${\hat{\Delta }}_{adj}$ in (Equation 2)

(9) $$\begin{array}{l} {\hat{\Delta }}_{adj}=\left({\hat{\mu }}_{i}^{*}-{\hat{\mu }}_{i}\right)-\left({\hat{\mu }}_{i\prime }^{*}-{\hat{\mu }}_{i\prime }\right)=\left({\hat{\mu }}_{i}^{*}-{\hat{\mu }}_{i\prime }^{*}\right)-\left({\hat{\mu }}_{i}-{\hat{\mu }}_{i\prime }\right)={\hat{\Delta }}_{PTM}-{\hat{\Delta }}_{protein} \end{array}$$

Assuming that the sources of variation in the summaries of modified features that are unexplained by the model are independent from the sources of variation in the summaries of modified features, the standard error $\hat{SE}\left({\hat{\Delta }}_{adj}\right)$ is obtained by combining the standard errors from the two model fits

(10) $$\begin{array}{l} \hat{SE}\left({\hat{\Delta }}_{adj}\right)=\sqrt{\hat{SE}{\left({\hat{\Delta }}_{PTM}\right)}^{2}+\hat{SE}{\left({\hat{\Delta }}_{protein}\right)}^{2}} \end{array}$$

For example, in the simple case of Figure 2 with J=2 replicates, where ${\hat{\sigma }}_{PTM}^{2}$ and ${\hat{\sigma }}_{Protein}^{2}$ are respectively the estimates of the error variance for the PTM and protein model described in (Equation 7), the standard error is calculated as

(11) $$\begin{array}{l} \hat{SE}\left({\hat{\Delta }}_{adj}\right)=\sqrt{\hat{SE}{\left({\hat{\Delta }}_{PTM}\right)}^{2}+\hat{SE}{\left({\hat{\Delta }}_{protein}\right)}^{2}}=\sqrt{\frac{1}{J}{\hat{\sigma }}_{PTM}^{2}+\frac{1}{J}{\hat{\sigma }}_{protein}^{2}} \end{array}$$

The estimated standard error is larger than the standard errors associated with each individual feature type, reflecting the combined uncertainty in the two estimates. Finally, the degrees of freedom associated with (Equation 10) are obtained *via* the Satterthwaite approximation (23, 37)

(12) $$df\left(\hat{SE}\left({\hat{\Delta }}_{adj}\right)\right)={\left(\hat{SE}{\left({\hat{\Delta }}_{PTM}\right)}^{2}+\hat{SE}{\left({\hat{\Delta }}_{protein}\right)}^{2}\right)}^{2}/\left(\frac{\hat{SE}{\left({\hat{\Delta }}_{PTM}\right)}^{4}}{df\left(\hat{SE}\left({\hat{\Delta }}_{PTM}\right)\right)}+\frac{\hat{SE}{\left({\hat{\Delta }}_{protein}\right)}^{4}}{df\left(\hat{SE}\left({\hat{\Delta }}_{protein}\right)\right)}\right)$$

To test the null hypothesis in (Equation 2), the test statistic ${\hat{\Delta }}_{adj}/\hat{SE}\left({\hat{\Delta }}_{adj}\right)$ is compared with the Student distribution with the degrees of freedom in (Equation 12). The *p*-values of the comparison are adjusted for multiple testing using the approach by Benjamin and Hochberg (38).

### Sample Size Calculation for Future PTM Experiments

The statistical framework in MSstatsPTM enables sample size calculation for future experiments studying changes in PTM. The procedure has been described in general in (23) and for protein significance analysis specifically in (39). It requires us to specify the desired levels of the following quantities: (a) q, the false discovery rate (FDR) of detecting differential abundance, (b) β, the average Type II error rate, (c) $\Delta_{adj}$, the minimal log_2_-fold change in adjusted PTM abundance of interest, (d) $m_{0}/\left(m_{0}+m_{1}\right)$, the fraction of truly differentially modified PTM sites in the comparison, and (e) $\sigma_{PTM}^{2}$ and $\sigma_{protein}^{2}$, the anticipated variances associated with the pairwise comparisons of conditions for the modified and unmodified protein summaries, respectively. Typically, these variances are estimated from an existing experiment, conducted with the same biological material and measurement workflow.

Given the above quantities and assuming a balanced design that allocates the same number of replicates J′ to both modified and unmodified profiling runs, the minimal number of replicates J′ for each of I conditions is chosen to bound the variance of the estimated log_2_-fold change $SE^{2}\left(\Delta_{adj}\right)\text{:}$

(13) $$\begin{array}{l} SE{\left(\Delta_{adj}\right)}^{2}=\left[\frac{2}{J\prime }\left(\sigma_{PTM}^{2}+\sigma_{protein}^{2}\right)\right]\leq {\left(\frac{\Delta_{adj}}{z_{1-\beta }+z_{1-\alpha /2}}\right)}^{2} \end{array}$$

where

(14) $$\begin{array}{l} \alpha =\left(1-\beta \right)\cdot \frac{q}{1+\left(1-q\right)\cdot m_{0}/m_{1}} \end{array}$$

and $z_{1-\beta }$ and $z_{1-\alpha /2}$ are the $100{\left(1-\beta \right)}^{th}$ and the $100{\left(1-\alpha /2\right)}^{th}$ percentiles of the standard normal distribution. Solving for J′, the number of biological replicates per condition is

(15) $$\begin{array}{l} J\prime \geq \frac{\left(2\sigma_{PTM}^{2}+2\sigma_{protein}^{2}\right){\left(z_{1-\beta }+z_{1-\alpha /2}\right)}^{2}}{\Delta_{adj}^{2}} \end{array}$$

The numerator has two sources of variation, reflecting a larger uncertainty in the adjusted calculation. Therefore, the adjustment typically requires a larger sample size to gain the same sensitivity as the unadjusted estimation.

In (Equation 15), we calculated the required sample size; however, in some cases, the statistical power of an experiment may be of interest. In this case, (Equation 13) is still used, however the number of replicates, J′ , is fixed and false discovery rate, q, is solved for.

### Implementation of MSstatsPTM

The implementation of the open source R package MSstatsPTM is overviewed in Fig. 3. By leveraging the implementations in MSstats and MSstatsTMT, the proposed approach is versatile. It is applicable to a wide variety of experimental designs, including group comparison, paired designs, time course designs, and unbalanced designs. It is applicable to label-free data acquisitions, such as DDA, DIA, and selected reaction monitoring, and label-based data acquisitions such as TMT. It can model experiments where the experimental designs for PTM profiling and global proteome profiling vary in properties such as number of biological replicates, data acquisition strategies, and runs.

MSstatsPTM takes as input lists of identified and quantified spectral features, produced by spectral processing tools such as MaxQuant, Progenesis, or Spectronaut (Step 3 of Fig. 3). Conversion is performed separately for the runs enriched in modified peptides and separately for the global profiling runs. We require the processing tools to identify the modification site (*i.e.*, the amino acid in the protein sequence where the modification occurred). This will generally include the amino acid abbreviation, plus its number in the protein sequence. For example, a modification on a 70th amino acid in the sequence, serine should be marked as “S70”. Occasionally the outputs of data processing tools only include the peptide sequence with the modified amino acid highlighted, without indicating the location in the protein sequence. For these cases, MSstatsPTM includes functionality for identifying the location, given the modified peptide sequence and a FASTA file with the entire protein sequence. The converters output the modified spectral features $y_{ijk}^{*}$, and the corresponding unmodified features $y_{ijk}$ in the format required for summarization.

The next step is PTM/protein summarization using the *dataSummarizationPTM*() function (Step 4 of Fig. 3). Summarization is performed separately for the PTM features and for the features representing the unmodified portion of the protein. When summarizing the PTM, modified peptide features that span the same modification site are summarized together. Peptides that include multiple modifications are not included in the single modification summarization and are grouped separately. The unmodified protein summarization is performed as discussed above for MSstats. When summarizing the unmodified protein features, the package optionally imputes missing values using an Accelerated Failure Time model (40). When summarizing the modified features, missing value imputation is also possible but should be performed with care. PTMs generally exhibit low feature counts and may be missing due to reasons other than low abundance. These issues can violate the assumptions underlying the imputation and lead to numerically unstable results. The outputs of this step are the run-level summaries for the modified ${\hat{y}}_{ij}^{*}$, and unmodified ${\hat{y}}_{ij}$, features.

Separate statistical models are fit to both feature summaries using the *groupComparisonPTM*() function (Step 5 of Fig. 3). MSstats or MSstatsTMT models are leveraged to automatically reflect the experimental design and the data acquisition. If the base model is not applicable for a particular PTM or protein*, e.g*., due to missing data, a simplified model is fit. The output of the models are the estimates ${\hat{\Delta }}_{PTM}$ and ${\hat{\Delta }}_{protein}$, and their standard errors $\hat{SE}\left({\hat{\Delta }}_{PTM}\right)$ and $\hat{SE}\left({\hat{\Delta }}_{protein}\right)$.

After modeling, the model for the modified peptides is adjusted for changes in the abundance of the unmodified portion of the protein, using the methods described above. Modification sites which lack corresponding global profiling information cannot be adjusted for changes in protein abundance. In this case, the implementation reverts to testing the null hypothesis in (Equation 1) using the statistical methods seen in MSstats, applied separately to each modified peptide. The final output is the estimate ${\hat{\Delta }}_{adj}$ and its standard error $\hat{SE}\left({\hat{\Delta }}_{adj}\right)$.

Finally, the statistical models can be used to calculate the required sample size for future PTM experiments using the *designSampleSizePTM*() function (Step 6 of Fig. 3).

In addition to the above functionalities, the implementation includes visualizations for quality control, *dataProcessPlotsPTM()*, and assessment of the quality of model fit, *groupComparisonPlotsPTM()*.

The implementation relies on functionalities from the R packages MSstats (16) and MSstatsTMT (17), which in turn rely on the R packages lme4 (41) and lmerTest (42). MSstatsPTM is available on Bioconductor, http://www.bioconductor.org/packages/release/bioc/html/MSstatsPTM.html, and Github, https://github.com/Vitek-Lab/MSstatsPTM.

### Evaluation

#### Evaluation Criteria

We compared the performance of MSstatsPTM to that of Limma and ANOVA, both before and after adjusting for changes in the unmodified portion of the protein. Since Isobar-PTM is only applicable to experiments with TMT labeling, it could not be applied to the datasets with known ground truth in this manuscript and was therefore excluded from the comparisons.

MSstatsPTM before adjusting for changes in the unmodified portion of the protein corresponds to base MSstats or MSstatsTMT, modeled on the peptide level as described in supplemental Fig. S1, as appropriate for the experimental design. MSstatsPTM with the adjustment described in Statistical Methods in MSstatsPTM was used without imputing missing values and without Empirical Bayes moderation.

Unadjusted ANOVA, *i.e.*, ANOVA before adjusting for changes in the unmodified portion of the protein, was as in (Equation 4). Adjusted ANOVA, *i.e.*, ANOVA with the adjustment was modeled as in (Equation 6). Finally, unadjusted Limma used the same model formula in (Equation 4), while including a moderated variance estimation. Adjusted Limma was modeled as in (Equation 6) including moderated variance estimation. All the evaluations were done at the FDR-adjusted *p*-value cutoff of q=.05. More details are in Supplementary Sec. 4.

We evaluated MSstatsPTM on simulated and spike-in datasets with known ground truth in terms of true positives (TPs), FPs, true negatives (TNs), and false negatives (FNs) differentially abundant PTMs. The TPs were defined as PTMs with changes distinct from the overall changes in abundance of the unmodified portion of the protein. The TNs were defined as PTMs which, after accounting for the changes in the overall protein abundance, were not differentially abundant. Additional summaries were performed including accuracy, recall, and positive predictive value (PPV)/empirical false discovery rate (eFDR) as described in (Equation 16).

(16) $$\begin{array}{l} Accuracy=\frac{TP+TN}{TP+TN+FP+FN},Recall=\frac{TP}{TP+FN} \end{array}$$

$$1-PPV=\frac{FP}{TP+FP}=empirical\;False\;Discovery\;Rate\;\left(eFDR\right)$$

For biological experiments with unknown ground truth, we compared the differentially abundant PTMs with and without adjusting for changes in unmodified protein abundance.

### Protein-Level Adjustment Was Required to Control eFDR in Differentially Abundant PTM

**Fig. 4:**
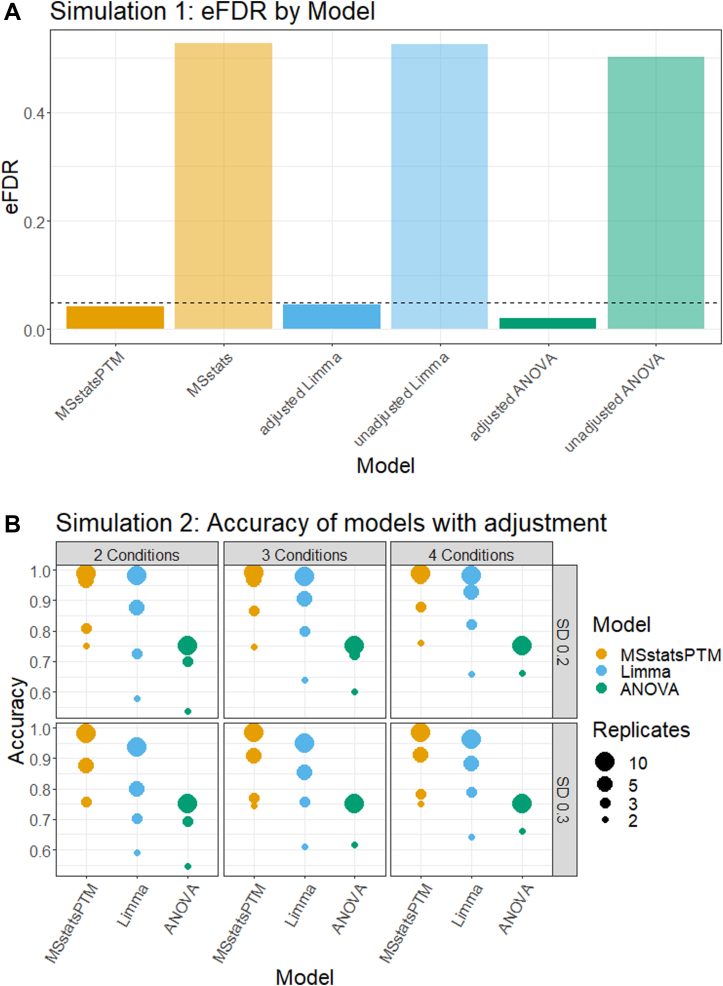
**Computer simulations**. *A,* dataset 1, clean simulation, analyzed to control eFDR at most 5% (*horizontal line*). The methods not accounting for the confounding between changes in PTM and overall changes in protein abundance produced exceedingly high numbers of false positives. In contrast, the methods accounting for the confounding correctly calibrated the proportion of false positive differentially abundant PTM. *B,* dataset 2, a noisy simulation, which included limited feature observations and missing values. MSstatsPTM had higher accuracy than ANOVA and Limma given the same noise, number of biological replicates, and number of conditions. eFDR, empirical false discovery rate; PTM, posttranslational modification.

Fig. 4*A* summarizes the eFDR reported on Computer Simulation one dataset by MSstatsPTM, adjusted ANOVA, adjusted Limma and base MSstats, unadjusted ANOVA, and unadjusted Limma methods. The simulation mimicked a “clean” label-free group comparison experiment, not compromised by issues such as deviations from model assumptions, missing values, and outliers. All the analyses were performed to control the eFDR at most 5%. Yet, even under these favorable circumstances, the models that did not adjust for confounding from changes in overall protein abundance produced an excessive number of FPs. The versions of the models that accounted for the confounding produced error rates that were much better calibrated at the desired level.

### In Noisy Simulations, MSstatsPTM More Accurately Detected Differentially Abundant PTM

Fig. 4*B* summarizes the overall accuracy of methods adjusting for changes in abundance of the unmodified portion of the proteins in Computer Simulation 2. The simulation mimicked a more realistic label-free group comparison experiment, including low counts of modified features and missing values. We evaluated the impact of low *versus* high noise of the number of biological replicates and of the number of conditions. For low noise, MSstatsPTM outperformed the existing methods across all conditions and number of replicates, with near 100% accuracy when the replicates were high. As the noise increased the accuracy of all the methods decreased, however MStatsPTM still outperformed the existing methods. The difference was primarily due to two reasons. First, the ratio-based summarization approach used by Limma and ANOVA requires measurements for both the PTM and unmodified protein. In contrast, MSstatsPTM can leverage the information in the PTM or unmodified portion of the protein if one of the two is missing. On average over all simulations, 3.94% of the total unmodified protein run summarizations used by MSstatsPTM were discarded by Limma and ANOVA due to missing PTM data. No PTM run summarizations were discarded due to missing protein data. Second, the robust Turkey Median Polish summarization in MSstatsPTM was more resistant to outliers.

Supplemental Fig. S2 further compares the fold change estimation across all modified peptides. MSstatsPTM showed a tighter distribution of estimated fold changes around the true fold change. Specifically, the interquartile range (IQR) of the estimated fold change for MSstatsPTM was on average 32.5% smaller than Limma and ANOVA’s IQR. While the mean of the estimated fold changes was generally correct for all the methods, the proposed approach correctly estimated the fold change more often across all PTMs.

### In the Label-Free Benchmark Experiment, MSstatsPTM Had a Higher Sensitivity

**Fig. 5:**
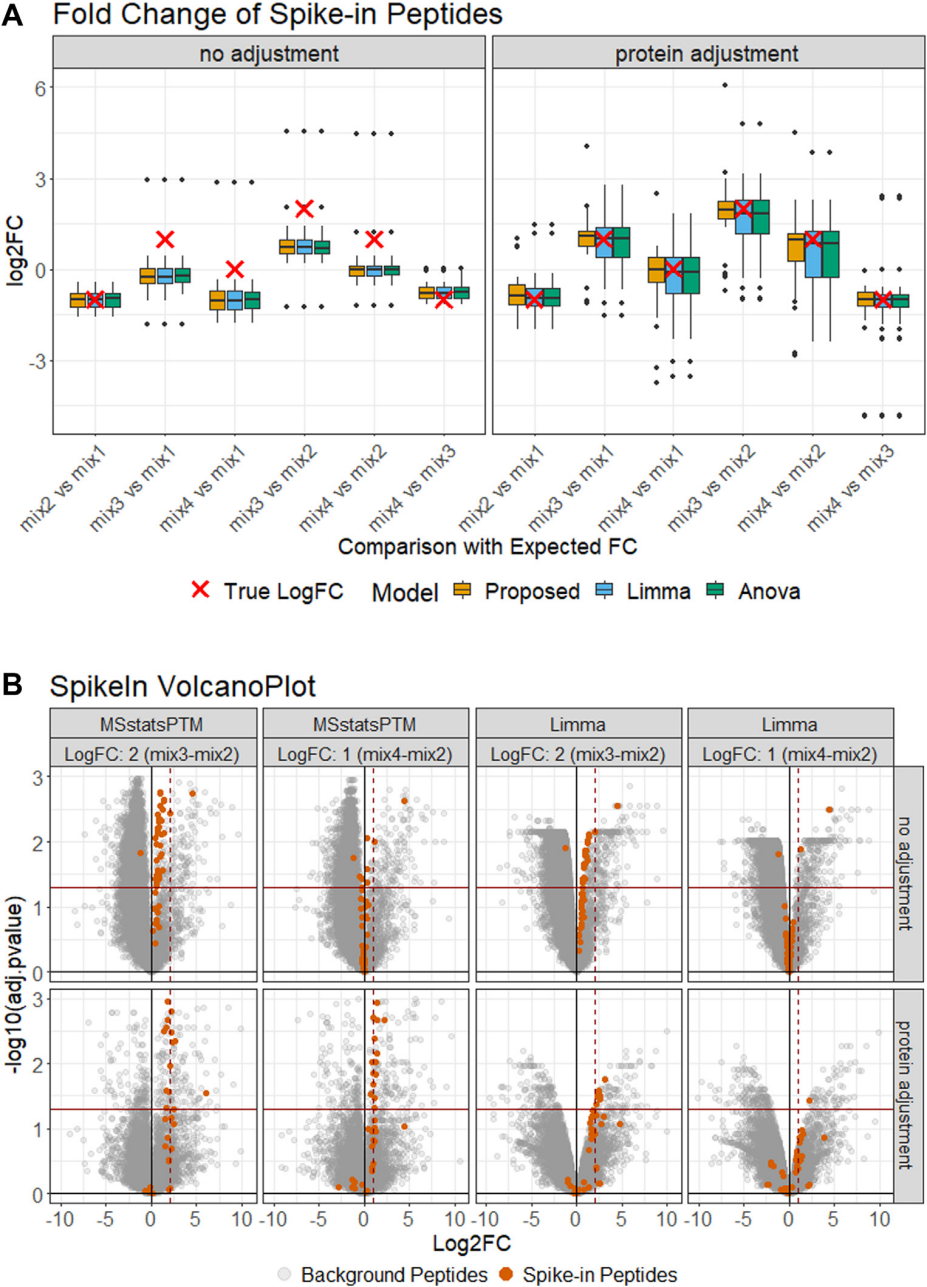
**Dataset 3: Spike-in benchmark—ubiquitination—label-free**. *A,* the distribution of spike-in peptides log_2_-fold change estimated by MSstatsPTM, Limma, and ANOVA with and without adjustment. The expected log_2_-fold change is highlighted by a *red* ‘X’. Protein adjustment removed systematic differences from the expected log_2_-fold change in all models. *B,* the statistical results of MSstatsPTM and Limma modeling the mix3-mix2 and mix4-mix2 comparisons before and after adjustment. The solid horizontal line shows the adjusted *p*-value cutoff of 0.05. The *solid vertical line* shows log_2_-fold change of 0. The *dashed vertical line* shows the expected log_2_-fold change of the spike-in peptides. Before adjustment, the spike-in peptides did not follow the expected log_2_-fold change but were more in line with expectation after adjustment.

Fig. 5*A* summarizes the evaluation on the label-free spike-in group comparison experiment, for all the methods, with and without adjusting for changes in abundance of the unmodified portion of the protein. Without adjusting for changes in unmodified protein abundance, all the approaches incorrectly estimated the log_2_-fold change of the modified spike-in peptides. After adjustment, the estimation was generally in line with the ground truth for all methods; however, MSstatsPTM’s distribution of estimated fold changes was tighter. On average over all comparisons the IQR of the estimates by MSstatsPTM was 32.86% smaller than that by Limma and ANOVA. As seen in the previous section, the robust summarization by MSstatsPTM was particularly useful when the number of features was low. This was also the case in this experiment, with an average of 1.37 features per PTM. Additionally, the ratio-based summarization used by Limma and ANOVA discarded more data than in the simulations. The ratio-based summarization lost 9.63% of the unmodified protein and 1.94% of the PTM run summarizations used by MSstatsPTM. This additional information lead MSstatsPTM to a more accurate fold change estimation and better calibrated variance.

Fig. 5*B* details the detection of differentially abundant PTM for MSstatsPTM and Limma, with and without the adjustment for changes in abundance in the unmodified portion of the protein, for the mix3-mix2 and mix4-mix2 comparisons. As above, the log_2_-fold changes of the spike-in peptides were only correctly estimated when accounting for changes in the unmodified protein abundance. Additionally, the background peptides, serving as the null model, show many FPs before adjustment. After adjustment, the extent of FPs substantially decreased. Specifically, for MSstatsPTM, the number of FPs went from 20.88% to 1.84% after the adjustment and for Limma went from 26.04% to 1.18%. While the proposed method and Limma both correctly estimated the fold change of the spike-in peptides, using Limma resulted in many large-adjusted *p*-values and lower sensitivity. This was mainly due to Limma estimating a higher variance, even when including variance moderation. On average, over all the PTMs in this experiment, the variance components estimated by Limma were 35.7% larger than for MSstatsPTM. Volcano plots for all methods and comparisons can be seen in Supplementary Section 4.2.

### In Two Biological Experiments With TMT Labeling, MSstatsPTM Corrected for Confounding Between Changes in the PTM and Changes in the Unmodified Protein

**Fig. 6:**
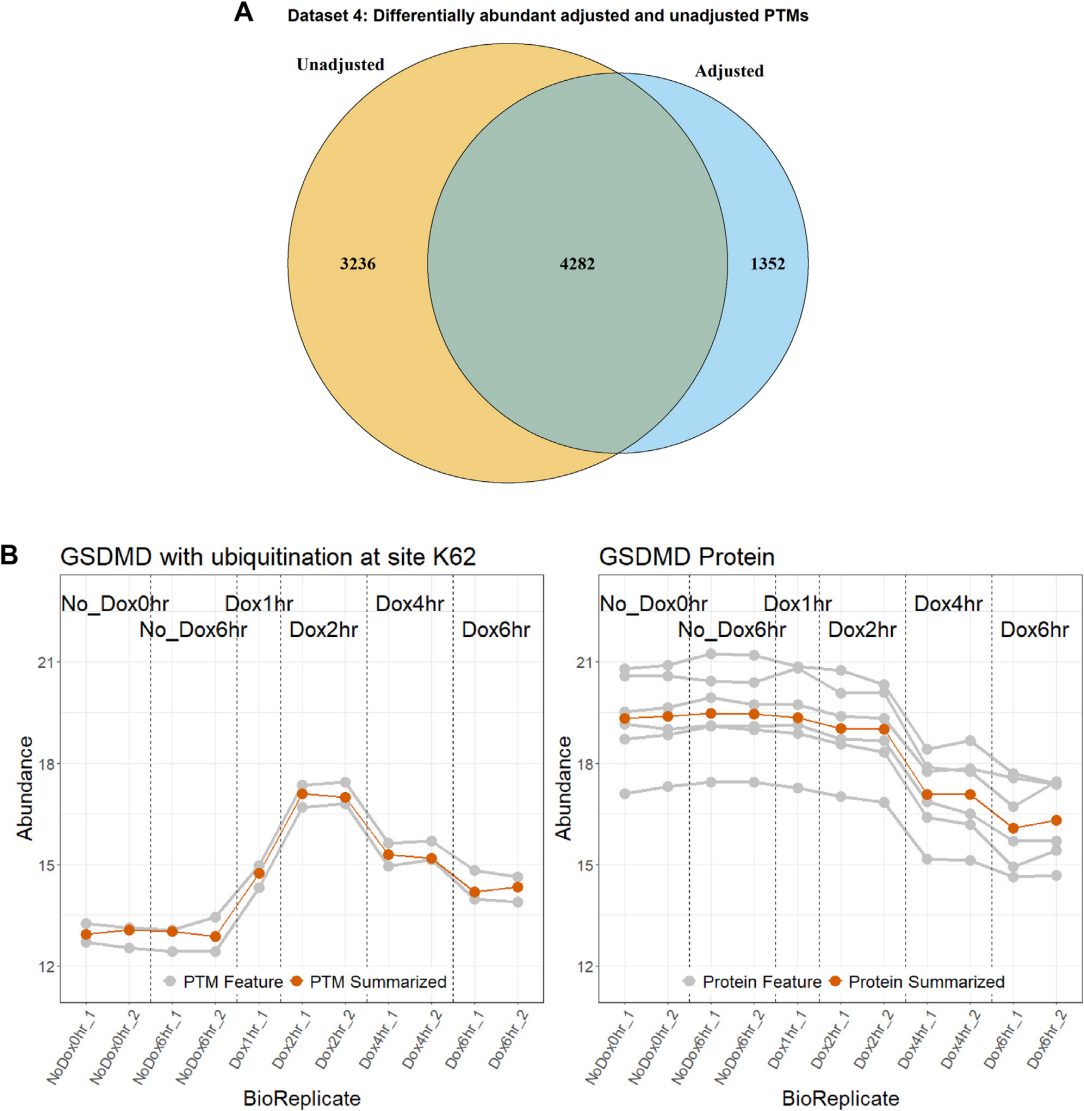
**Dataset 4: Human—ubiquitination—1mix-TMT, analysis with MSstatsPTM**. *A,* the overlap of differential modified peptides for the PTM model with and without global protein level adjustment across all pairwise comparisons. *B,* comparing the profiling of protein GSDMD with the ubiquitination at site K62. The individual PTM and protein features are shown in *gray*, while the summarization is highlighted in *red*. GSDMD, Gasdermin D; PTM, posttranslational modification; TMT, tandem mass tag.

Fig. 6*A* summarizes the results of Dataset 4: Human—Ubiquitination—1mix-TMT in terms of number of differentially abundant PTMs before and after adjustment. Adjusting for changes in abundance in the unmodified portion of the protein caused fewer PTMs to be detected as differentially abundant. A question is whether this was due to adjustment in the log_2_-fold estimation or to an increase in standard errors during the adjustment (Equation 10). To check that, we considered modified peptides for which the adjusted log_2_-fold change was within 10% of the unadjusted log_2_-fold fold change, but which lost statistical significance after the adjustment. In the case of Dataset 4, only one PTM became nondifferentially abundant due to an increase in standard error. In other words, the decrease in differentially abundant PTMs was primarily due to removing the confounding with global protein abundance and not to larger variance estimates.

Cases where true changes in PTMs were masked by changes in abundance in the unmodified portion of the protein were less frequent. One such case is in Fig. 6*B*. Luchetti *et al*. (20) showed that GSDMD was actively degraded when IpaH7.8 expression was induced by Dox treatment. Our reanalysis confirmed that the GSDMD protein was downregulated when Dox treatments reached the 4 and 6 h marks. Conversely, ubiquitination of GSDMD at site K62 upregulated abundance between the same conditions. This upregulation was originally confounded by the downregulation of unmodified GSDMD and made the modification appear to have little change between no Dox and Dox 4 and 6 h conditions. The proposed approach accounted for this confounding and the modification was detected as differentially abundant, with a log_2_-fold change of 2.79 between the Dox 1 h and Dox 4 h conditions (supplemental Fig. S6). The change in PTM abundance would have been challenging to observe without the proposed approach. Indeed, the modification contradicts the previous research focusing on global profiling of the GSDMD protein (20).

**Fig. 7:**
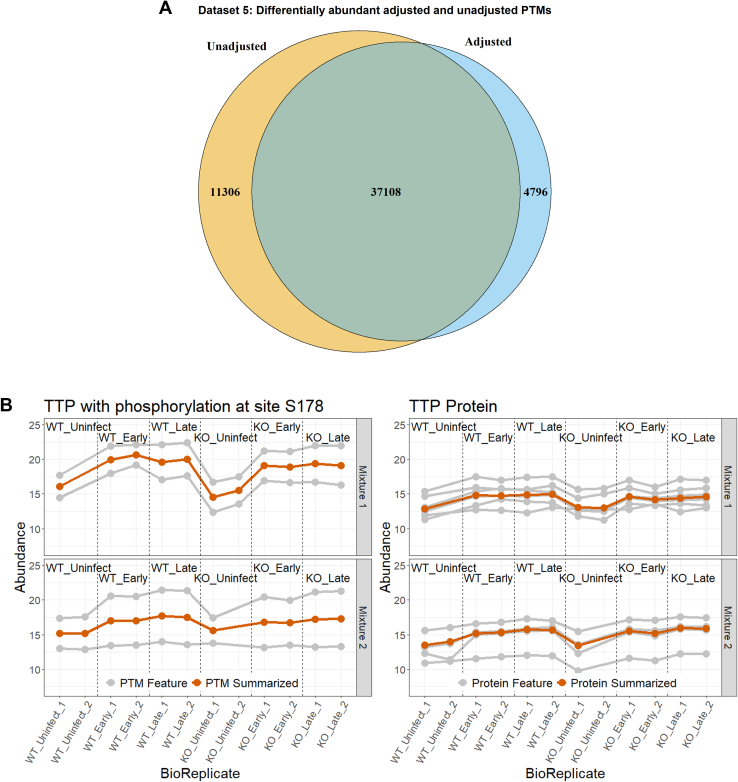
**Dataset 5: Mouse—phosphorylation—2mix-TMT, analysis with MSstatsPTM**. *A,* the overlap of differential modified peptides for the PTM model with and without global protein level adjustment across all pairwise comparisons. *B*, comparing the profiling of protein TTP with the phosphorylation at site S178. The individual PTM and protein features are shown in *gray*, while the summarization is highlighted in *red*. The plots are separated according to TMT Mixtures 1 and 2. PTM, posttranslational modification; TMT, tandem mass tag.

Fig. 7*A* illustrates a similar result for Dataset 5: Mouse—Phosphorylation—2mix-TMT. Adjusting for changes in abundance in the unmodified portion of the protein caused fewer PTMs to be detected as differentially abundant. As above, we checked whether this change was due to fold change adjustment or an increased standard error. In the case of Dataset 5548 PTMs were not detected as differential abundant due to an increased standard error. This corresponded to 3.4% of all the PTMs that became nondifferentially abundant.

**Supplemental Figure 7a:**
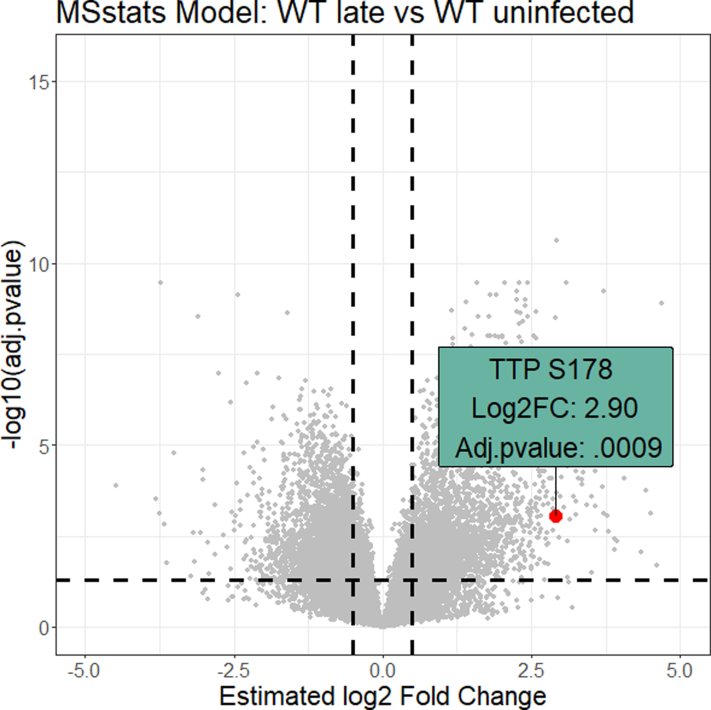


Fig. 7*B* shows an opposite case where the protein exhibits a change in abundance while the modification does not. Before adjusting for changes in the unmodified protein, the modification at S178 of protein TTP was shown to be differentially abundant between WT uninfect and WT late, with a log_2_-fold change of 2.9. However, the unmodified protein was shown to contribute 69.5% of this change, while the modification only accounted for 30.5% after adjustment (supplemental Fig. S7). This caused the modification to lose differential abundance.

### In Label-Free Experiment Without a Separate Global Profiling Run, MSstatsPTM Eliminated the Confounding Due to Changes in the Unmodified Protein, Albeit Less Effectively Than in the Presence of a Global Profiling Run

Unlike the other datasets in this manuscript, Dataset 6: Human—Ubiquitination—Label-free had no unmodified global profiling run. Therefore, after peptide identification and quantification, data from unmodified peptides were used separately in place of a global profiling run. This resulted in a sparse coverage of the modified features by the unmodified protein counterparts. Of the 10,799 identified ubiquitination sites, only 4526 had features from unmodified portion of the same protein. A PTM without features from the unmodified portion of the protein could not be adjusted for the confounding. Additionally, the lack of a separate global profiling run resulted in low feature counts and noisier measurements of the unmodified peptides as compared to the other experiments (Table 1).

**Fig. 8:**
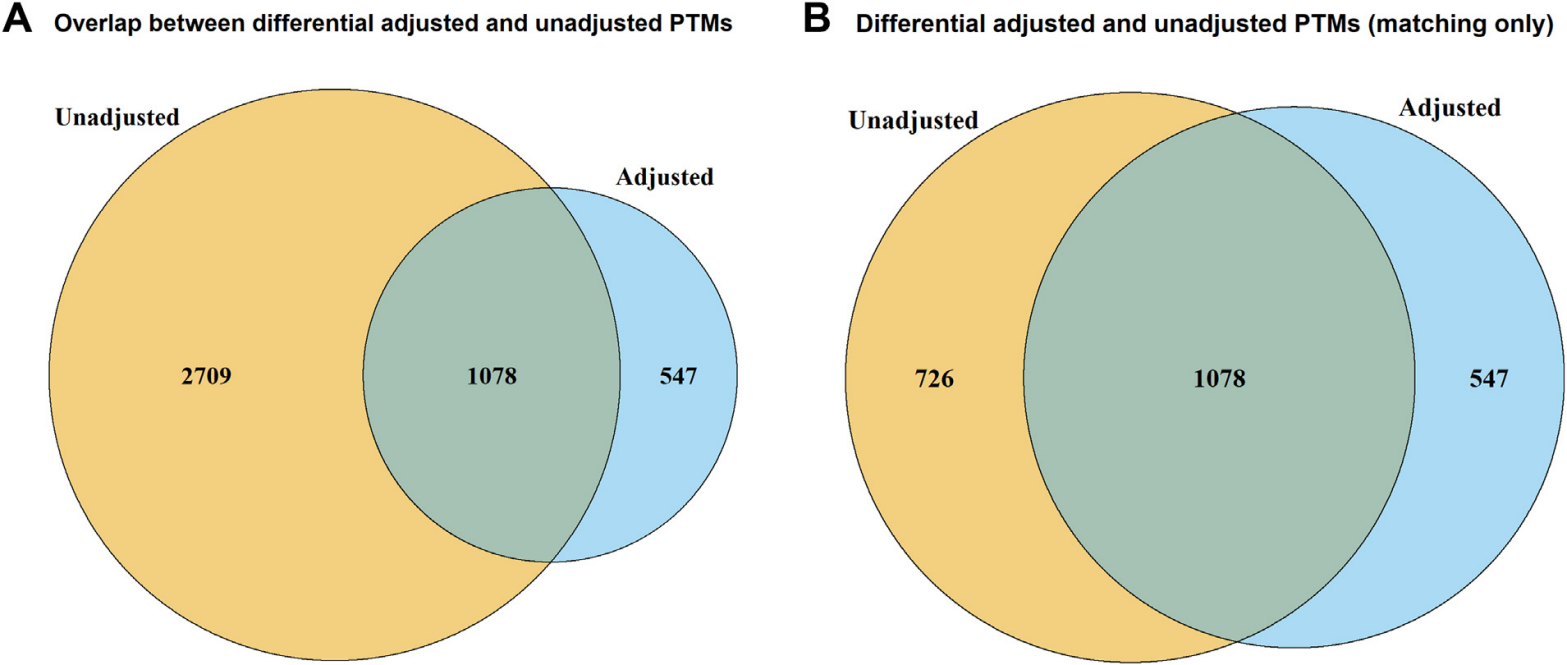
**Dataset 6: Human—ubiquitination—label-free no global profiling run, analysis with MSstatsPTM**. *A*, the overlap of differential modified peptides for the PTM model with and without global protein level adjustment across all comparisons, including all measured PTMs. *B,* The overlap of differential modified peptides with and without global protein level adjustment, including only PTMs for which features from the unmodified portion of the same protein were also available.

Fig. 8 shows the number of differentially abundant PTMs before and after adjustment. After adjusting for changes in abundance in the unmodified portion of the protein, the number of differentially abundant PTMs decreased. However, this was mainly due to the lack of a global profiling run. The decrease in differentially modified PTMs was smaller among PTMs with available unmodified protein counterparts. As above, we tested if this drop in differentially abundant PTMs was due to an increase in standard error. Here only 25 PTMs lost differential abundance due to increased standard error.

### Noisy PTM Measurements Benefited From Additional Biological Replicates

Sample size calculations and statistical power analyses help evaluate the benefit of additional biological replicates, in particular when measurements are noisy. Since the proposed approach does not have the restriction of the equal number of modified and unmodified global profiling runs, it is also interesting to evaluate the interplay between the number of replicates of each type in presence of differing amounts of noise. In datasets 4 and 5, the variance of the PTMs was higher than that of the unmodified protein summaries, with median values of PTM variance of 0.45 and protein variance of 0.3. In dataset 6, the variance of the PTM and the unmodified protein summaries were comparable, with a median variance of 0.45. The power analysis took as input these median variance values.

**Fig. 9:**
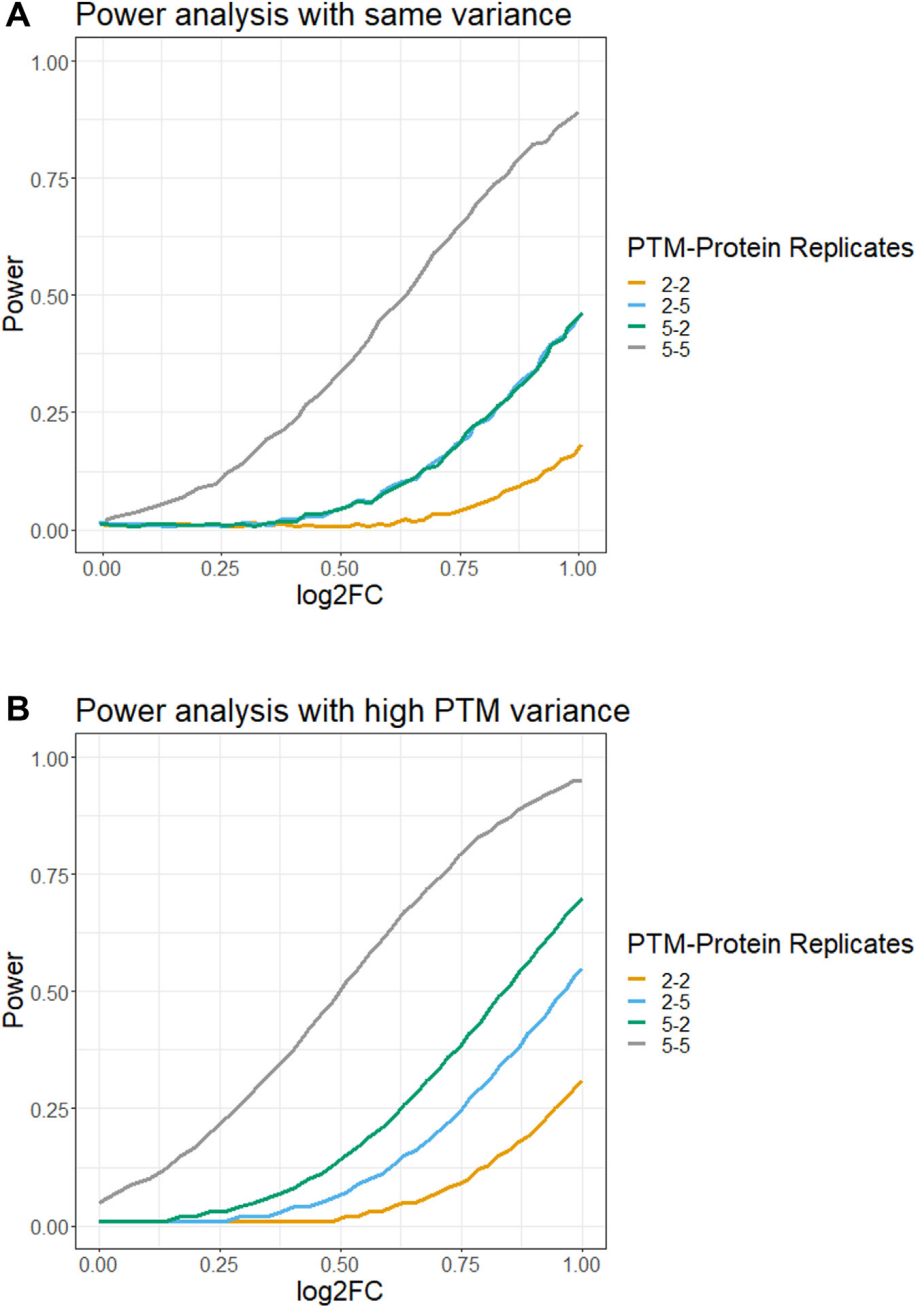
**Power analysis with MSstatsPTM, based on variance components from Datasets 4, 5, & 6**. Y-axis: statistical power, *i.e.,* the probability of rejecting the null hypotheses of no protein-adjusted change in a PTM between conditions, when in fact the change exists. X-axis: log_2_-fold change, after for adjusting for changes in the unmodified protein. *A*, the power of detecting differential abundant PTMs, when the modified features and the unmodified protein summaries have the same variance 0.45. *B,* the power of detecting differential abundant PTMs, when the modified features had a larger variance (0.45) as compared to the unmodified protein summaries (0.3). PTM, posttranslational modification.

Fig. 9 shows that regardless of the relative amount of variation, larger adjusted log_2_-fold changes and larger number of replicates enabled larger statistical power. When the variance of PTMs was equal to the variance of global protein profiles, it did not matter whether we increased the number of PTM runs or the number of unmodified protein runs. However, when the variance of the PTM summaries was higher than that of the unmodified protein summaries, allocating more biological replicates to the PTM profiles lead to a more efficient increase of statistical power.

## Discussion

We proposed a statistical modeling framework for detecting differentially abundant PTM and its implementation in MSstatsPTM. The proposed approach removes the confounding of changes in PTMs with changes in the unmodified protein summaries and can reveal modifications of interest that are otherwise entirely masked by changes in abundance in the unmodified portion of the protein. This is valuable, because many PTMs are often found nondifferentially abundant prior to the adjustment, and validating them manually to establish false negatives is generally unfeasible.

Our results show that MSstatsPTM is more accurate and more sensitive than the existing approaches. The gain is due to a more efficient use of the data and to a more accurate representation of the systematic and random variations. While the ratio-based approaches of ANOVA and Limma first consider differences within samples, MSstatsPTM first considers the differences between conditions. This enables a greater flexibility in terms of modeling complex designs, accounting for outliers and missing values and planning subsequent experiments.

The implementation of MSstatsPTM is a straightforward extension of MSstats and is therefore applicable to the full breadth of experiment types supported by MSstats. Although demonstrated here on DDA, it is also applicable to DIA, selected reaction monitoring, and parallel reaction monitoring acquisitions. Additionally, the approach can handle experiments that use different strategies for modified *versus* unmodified peptides, *e.g*., using label-free methods for unmodified peptides and TMT labeling for modified peptides or vice versa.

The proposed approach assumes that all the peptides are correctly mapped to the underlying proteins and PTM sites and that the features are informative of the protein and PTM abundances. However, multiple modification sites per peptide can confound the abundance of each PTM site. Changes in the unmodified peptide (as opposed to the unmodified protein) can also confound changes in PTM abundance. One potential solution is to quantify the abundance of peptides with one modification and use this to adjust the peptide with multiple sites to remove the confounding. However, this method would likely be challenged by scarcity of modified peptide features containing both a single and multiple modification sites. As the result, peptides with multiple modifications are currently beyond the MSstatsPTM scope.

Overall, MSstatsPTM balances accuracy and practicality and enables the analysis of complex experiments in high throughput. Future work is to carry out the inference and testing for not only the relative change of PTM abundance but also the fraction of the protein that is modified at the particular site (site occupancy, or stoichiometry) and attempt to remove the confounding of individual PTMs in peptides with multiple modifications.

## Data Availability

The datasets for the six experiments analyzed in this manuscript are available online. The spike-in and biological experiments raw data, processed data, and R code used for analysis are located on MassIVE.quant. The simulated data and code to generate the data are available on Github. Links to the containers are available in Table 1.

## Supplementary data

This article contains supplemental materials (18, 43, 44).

## Figures and Tables

**Supplemental Figure 2a:**
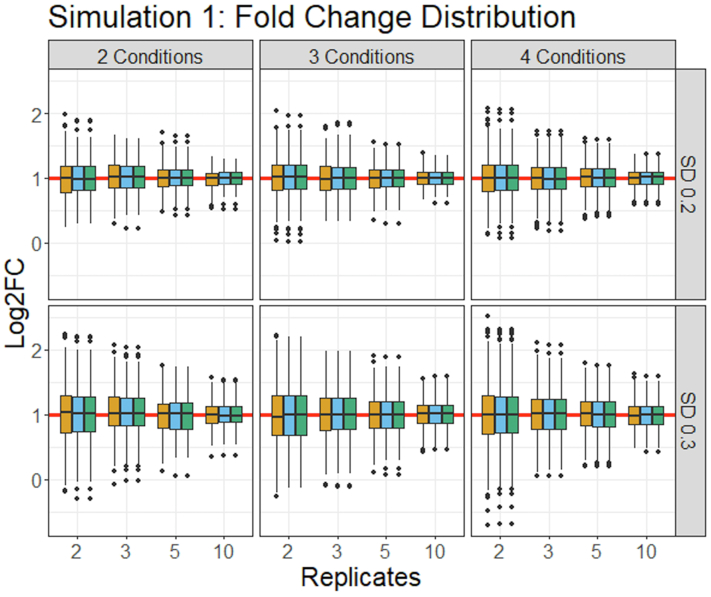


**Supplemental Figure 2b:**
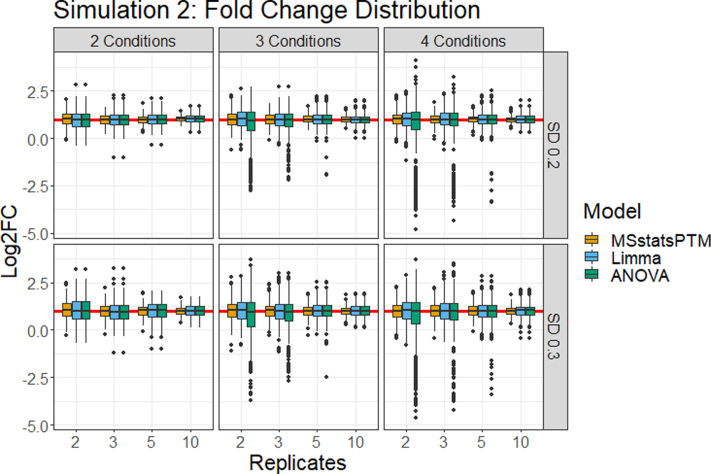


**Supplemental Figure 3:**
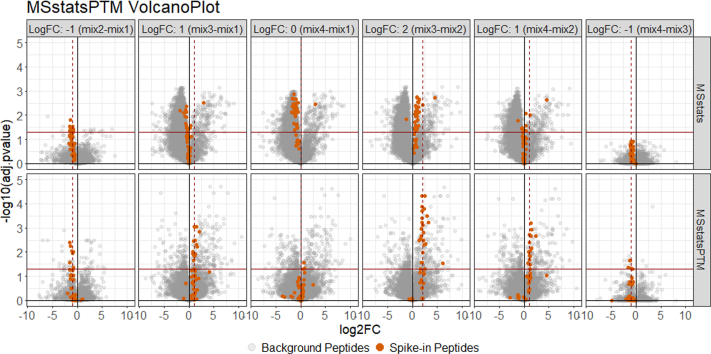


**Supplemental Figure 4:**
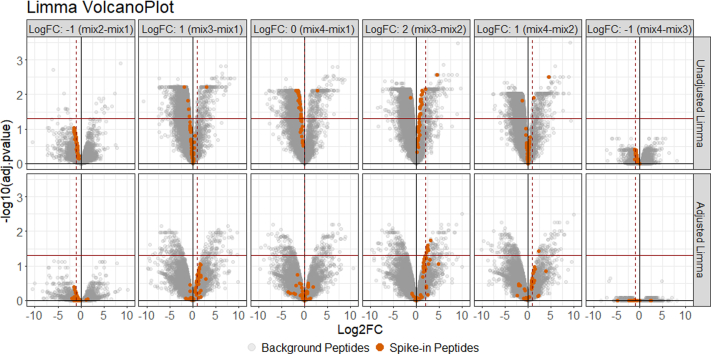


**Supplemental Figure 5:**
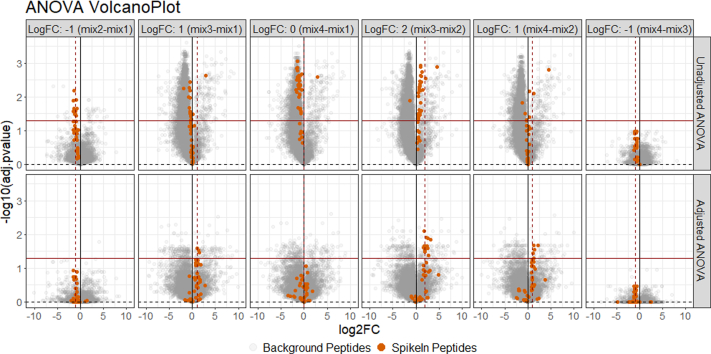


**Supplemental Figure 6a:**
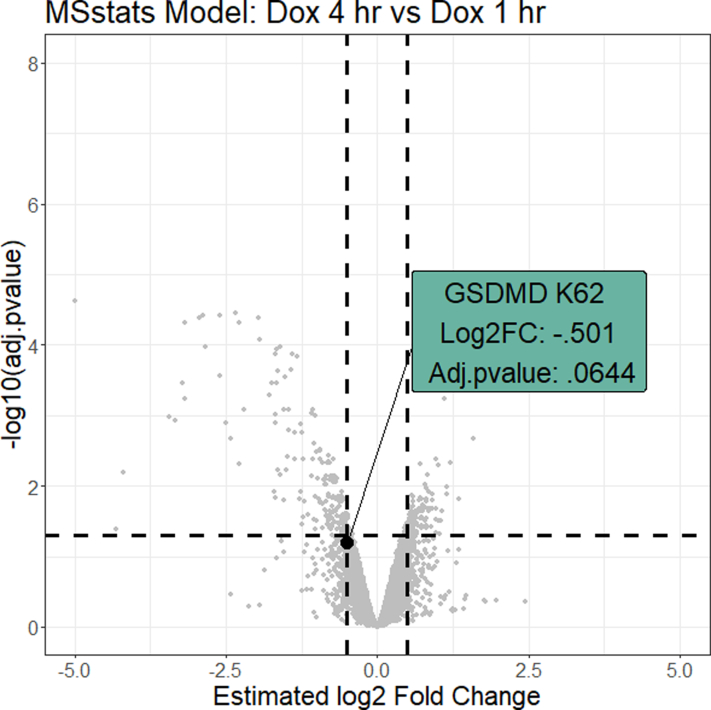


**Supplemental Figure 6b:**
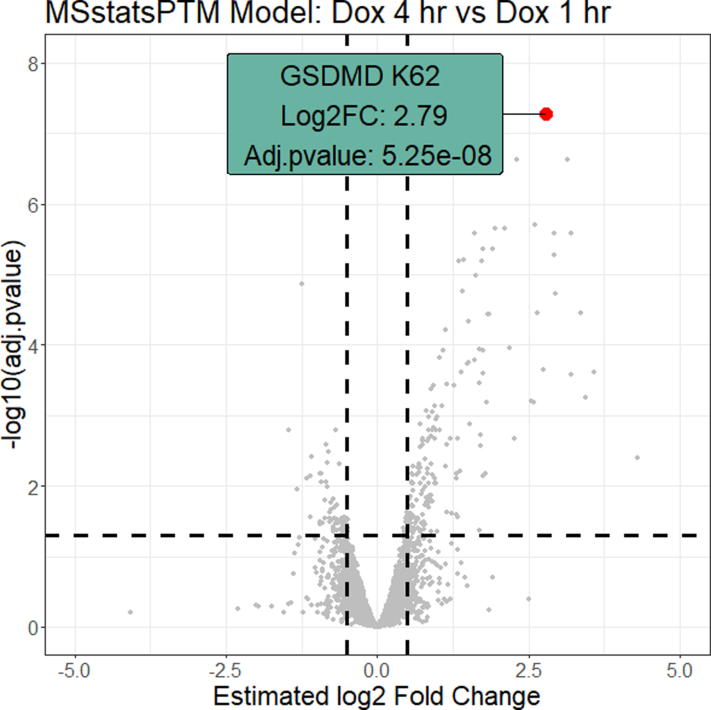


**Supplemental Figure 7b:**