# Supplementary material for: MSstatsPTM: Statistical Relative Quantification of Posttranslational Modifications in Bottom-Up Mass Spectrometry-Based Proteomics
Source: Mol Cell Proteomics. 2022 Dec 8;22(1):100477. doi: 10.1016/j.mcpro.2022.100477 (PMC9860394; doi:10.1016/j.mcpro.2022.100477)
Supplement: Supplemental data [file mmc1.pdf]

MSstatsPTM statistical relative quantification of post-translational modifications in global proteomics experiments

Supplementary Information

Devon Kohler[1], Tsung-Heng Tsai[2], Erik Verschueren[4], Ting Huang[1], Trent Hinkle[3], Lilian Phu[3], Meena Choi*[3], and Olga Vitek*[1]

[1]Khoury College of Computer Science, Northeastern University, Boston, MA, USA
[2]Department of Mathematical Sciences, Kent State University, Kent, OH, USA
[3]MPL, Genentech, South San Francisco, CA, USA
[4]ULUA BV, Arendstraat 29, 2018 Antwerp, Belgium
*Corresponding Authors

Contents

1 Detailed methods for Dataset 3: SpikeIn benchmark 3

1.1 Sample preparation 3
1.2 Immunoaffinify enrichment of KGG peptides 3
1.3 Mass Spectrometry Analysis 3
1.4 Data analysis 3

2 Details of the proposed approach 5

3 Details on the generation of simulated datasets 6

3.1 Dataset 1 : Computer simulation 1 - Label-free 6
3.2 Dataset 2 : Computer simulation 2 - Label-free missing values and low features 6

4 Additional evaluation results 7

4.1 Simulated Datasets 1 and 2 7
4.2 Dataset 3 : SpikeIn benchmark - Ubiquitination - Label-free 8
4.3 Dataset 4 : Human - Ubiquitination - 1mix-TMT 10
4.4 Dataset 5 : Mouse - Phosphorylation - 2mix-TMT 12

1 Detailed methods for Dataset 3: SpikeIn benchmark

1.1 Sample preparation

HEK293 and E.coli cells were prepared separately according to the following protocol. Cells were lysed in denaturing buffer (8M urea, 20 mM HEPES pH 8.0, 1 mM sodium orthovanadate, 2.5 mM sodium pyrophosphate, and 1 mM β-glycerophosphate) and subjected to two rounds of microtip sonication (9W for 30 s) at cold temperature. Lysates were clarified via high speed centrifugation (18,000 x g, 15 min) to remove insoluble material, reduced (4.1 mM dithiothreitol, 60 min at 37°C) and alkylated (9.1 mM iodoacetamide, 15 min at room temperature). HEK293 (120 mg) and E.coli (40mg) protein lysates were separately taken forward for enzymatic digestion. Lysates were diluted 4-fold and subjected to overnight enzymatic digestion at 37°C with a combination of lysyl-endopeptidase (Wako) and sequencing grade trypsin (Promega), both at an enzyme to protein ratio of 1:100, the latter of which was added to the sample 3 h post incubation with the former. Resultant peptides were acidified with 20% TFA to a final concentration of 1%, clarified via high speed centrifugation (18,000 x g, 10 min) prior to desalting via Sep-pak C18 solid phase extraction (Waters). HEK293/E.coli peptide mixtures were subsequently prepared in the ratios as described and less than 1% of each of the mixed peptide samples was saved for global protein assessment. The peptide mixtures were then lyophilized for 48 h and reconstituted in 1X detergent containing IAP buffer (Cell Signaling Technology). Spike in peptides were added prior to proceeding with immunoaffinity enrichment.

1.2 Immunoaffinify enrichment of KGG peptides

Enrichment for peptides containing diglycine modified lysine residues (KGG) was performed on a MEA2 automated purification system (Phynexus) using 1 mL Phytips (Phynexus) packed with 20 μl ProPlus resin coupled to 200 μg of anti-KGG (Cell Signaling Technology) antibody. Immunoaffinity enrichment on the MEA2 was performed as follows. Phytip columns were equilibrated for 2 cycles (1 cycle = aspiration and dispensing, 0.9 ml, 0.5 mL/min) with 1 mL 1X IAP buffer prior to contact with peptides. Phytip columns were incubated with peptides for 16 cycles of capture, followed by 6 cycles of wash, 2X with 1 mL 1X IAP buffer and 4X with 1 mL water. Captured peptides were eluted with 60 μl 0.15% TFA in 8 cycles where the volume aspirated/dispensed was adjusted to 0.06 ml.

1.3 Mass Spectrometry Analysis

Samples were resuspended in solvent A (2% acetonitrile (ACN)/0.1% formic acid (FA)) and analyzed by liquid chromatography tandem mass spectrometry (LC-MS/MS) on an Orbitrap Lumos mass spectrometer (ThermoFisher) coupled to a Dionex Ultimate 3000 RSLC (ThermoFisher) employing an Aurora Series 25 cm x 75 um I.D. column (IonOpticks, Australia). Peptides were separated at a flowrate of 450 nL/minute over a 95 min dual stage linear gradient whereby solvent B (0.1% FA/98%ACN/2% water) was ramped from 2% to 35% over 85 min and then from 35% to 50% over 10 min with a total run time of 120 min. The mass spectrometer was operated in data dependent acquisition mode with MS1 precursor ions analyzed in the FT at 240,000 resolution with an AGC target of 1e6.MS2 was performed in the linear ion trap with a high-energy collision dissociation (HCD) normalized collision energy of 30%, an AGC target of 40,000, an isolation width of 0.7 m/z, and a 10 s dynamic exclusion window.

1.4 Data analysis

MS/MS spectra for the global proteome and ubiquitylated data sets were searched using the Mascot search algorithm (Matrix Sciences) against a concatenated target-decoy database comprised of the UniProt human and E. Coli protein sequence (version 2017/08), known contaminants and the reversed versions of each

sequence. For all datasets, a 50 ppm precursor ion mass tolerance was selected with tryptic specificity up to two missed cleavage. For the global proteome dataset a 0.8 Da fragment ion tolerance was selected. The global proteome datasets used a fixed modification of carbamidomethyl cysteine and methionine oxidation for variable modifications. For KGG (ubiquitin) dataset a 0.02 Da fragment ion tolerance was selected. The KGG dataset used methionine oxidation as well as ubiquitylation on lysine for variable modifications, carbamidomethyl cysteine for fixed modification. PSMs were filtered to a 1% peptide FDR at the run level using linear discriminant analysis (LDA) [1]. For PSMs passing the peptide and protein FDR filters within the ubiquitylated datasets, ubiquitylation site localization was assessed using a modified version of the AScore algorithm (cutoff=15) [2] and reassigned accordingly. Finally, reporter ion intensity values were determined for each dataset using Vista algorithm [3] with an isolation width of 0.7.

2 Details of the proposed approach

		Model	Estimated Log-fold change	Theoretical variance	Estimated variance	Degrees of freedom
**Label-free**<br>(y_{ij} is $\log_2$ intensity in Condition i and BioReplicate j)	**Group comparison**	$y_{ij} = \mu_i + \varepsilon_{ij}$<br>$\sum_{i=1}^{I} \mu_i = 0$<br>$\varepsilon_{ij} \sim iid\ N(0, \sigma^2)$	$\bar{Y}_{i.} - \bar{Y}_{i'.}$	$\frac{2\sigma^2}{I}$	$\frac{2J \sum_{i=1}^{I} (y_{ij} - \bar{y}_{i.})^2}{J(IJ - I)}$	$IJ - I$
	**Time course**	$y_{ij} = \mu_i + BioReplicate_j + \varepsilon_{ij}$<br>$\sum_{i=1}^{I} \mu_i = 0$<br>$BioReplicate_j \sim iid\ N(0, \sigma_J^2)$<br>$\varepsilon_{ij} \sim iid\ N(0, \sigma^2)$	$\bar{Y}_{i.} - \bar{Y}_{i'.}$	$\frac{2\sigma^2}{I}$	$\frac{2 \sum_{i=1}^{I} \sum_{i=1}^{J} (y_{ij} - \bar{y}_{i.} - \bar{y}_{j.} + \bar{y}_{..})^2}{J(I-1)(J-1)}$	$(I-1)(J-1)$
**TMT**<br>(y_{mij} is $\log_2$ intensity in Condition i and BioReplicate j from Mixture m)	**Group comparison**	$y_{mij} = \mu_i + Mixture_m + \varepsilon_{mij}$<br>$\sum_{i=1}^{I} \mu_i = 0$<br>$Mixture_m \sim iid\ N(0, \sigma_M^2)$<br>$\varepsilon_{mij} \sim iid\ N(0, \sigma^2)$	$\bar{Y}_{.i.} - \bar{Y}_{.i'.}$	$\frac{2\sigma^2}{MJ}$	$\frac{2J \sum_{i=1}^{I} \sum_{m=1}^{M} (y_{mij} - \bar{y}_{m..} - \bar{y}_{.i.} + \bar{y}_{...})^2}{MJ(MIJ - I - M + 1)}$	$MIJ - I - M + 1$
	**Time course**	y_{mij}<br>$= \mu_i + BioReplicate_{jm} + \varepsilon_{mij}$<br>$\sum_{i=1}^{I} \mu_i = 0$<br>$BioReplicate_{jm} \sim iid\ N(0, \sigma_J^2)$<br>$\varepsilon_{mij} \sim iid\ N(0, \sigma^2)$	$\bar{Y}_{.i.} - \bar{Y}_{.i'.}$	$\frac{2\sigma^2}{MJ}$	$\frac{2J \sum_{j=1}^{I} \sum_{m=1}^{M} (y_{mij} - \bar{y}_{mj.} - \bar{y}_{.i.} + \bar{y}_{...})^2}{MJ(I-1)(MJ-1)}$	$(I-1)(MJ-1)$

Figure S1: Different models that are fit depending on the experimental design (group comparison and time course) and quantification workflow (label-free versus TMT). The table shows the true values of the standard errors, along with their estimates and the associated degrees of freedom. The same formulas holds when comparing changes in PTM, or changes in the unmodified portion of the protein. When combining the two comparisons for an adjustment, the variance must be multiplied by 2.

3 Details on the generation of simulated datasets

3.1 Dataset 1 : Computer simulation 1 - Label-free

In the first simulation an experiment with many features per PTM and unmodified protein was created. Additionally this simulation contained no missing data.

- Mean of log-intensity: 25
- Standard deviations of log-intensities for modified and unmodified peptides: 0.2, 0.3
- Difference in PTM abundance between conditions: 0, 1., 2., 3.
- Difference in protein abundance between conditions: 0, 1., 2., 3.
- Number of replicates: 2, 3, 5, 10
- Number of conditions: 2, 3, 4
- Number of realizations: 1000
- Number of features per PTM: 10
- Number of features per unmodified protein: 10
- Missing data: no missing value

3.2 Dataset 2 : Computer simulation 2 - Label-free missing values and low features

In the second simulation we introduced limited feature observations per PTM as well as masking a portion of the observation to simulate missing values.

- Mean of log-intensity: 25
- Standard deviations of log-intensities for modified and unmodified peptides: 0.2, 0.3
- Difference in PTM abundance between conditions: 0, 1., 2., 3.
- Difference in protein abundance between conditions: 0, 1., 2., 3.
- Number of replicates: 2, 3, 5, 10
- Number of conditions: 2, 3, 4
- Number of realizations: 1000
- Number of features per PTM: 2
- Number of features per unmodified protein: 10
- Missing data: 20% of the observations for PTMs and Proteins were masked with NA at random

4 Additional evaluation results

4.1 Simulated Datasets 1 and 2

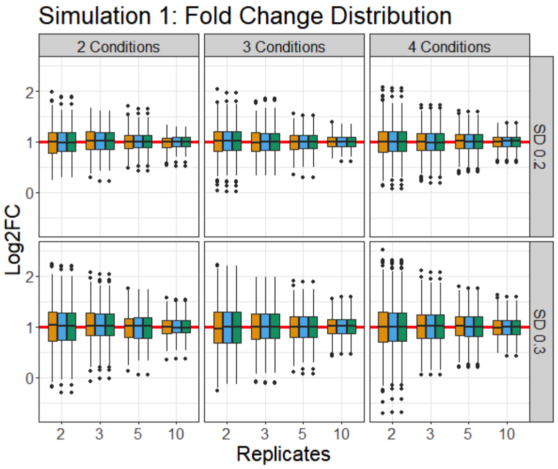

(a)

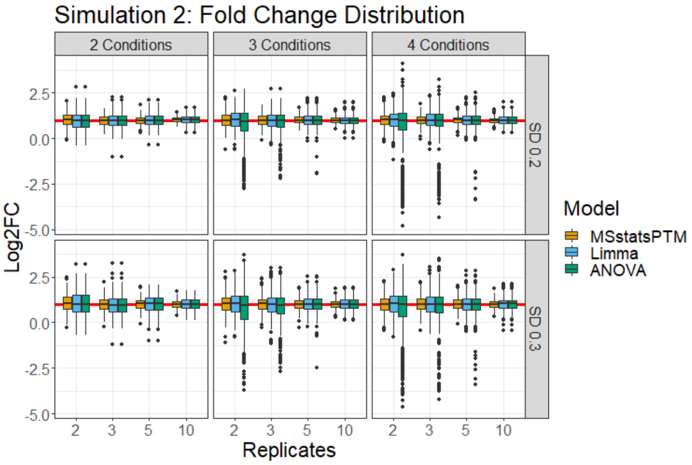

(b)

Figure S2: Simulated Datasets 1 and 2 : fold change distributions. All models are shown after adjusting for changes in unmodified protein abundance. (a) In Simulated Dataset 1 all considered methods correctly estimated the fold change between conditions, with a median fold change estimation of 1. The distributions around the median were also consistent across all methods. (b) In Simulated Dataset 2 all methods correctly estimated the fold change with a median log change of 1. *MSstatsPTM* in this simulation had a tighter distribution around the median. Both *Limma* and *ANOVA* showed a wider range around the fold change.

4.2 Dataset 3 : SpikeIn benchmark - Ubiquitination - Label-free

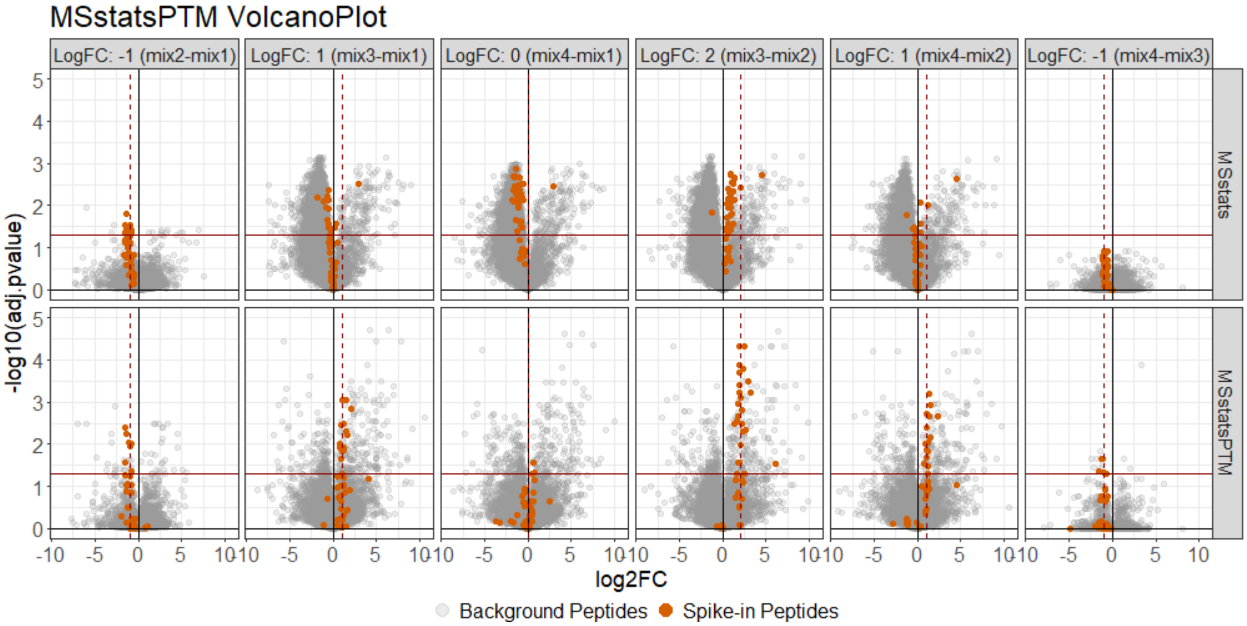

Figure S3: Dataset 3 : SpikeIn benchmark - Ubiquitination - Label-free. The modeling results of the *MSstatsPTM* both before and after adjustment. The unadjusted model is the basic version of *MSstats* before adjustment. The spike-in peptides are colored red and the background peptides are colored grey. All grey peptides are expected to not be detected as differentially abundant. The spike-in peptides (colored red) did not follow the expected log fold change before adjustment. After adjusting for changes in overall protein abundance the spike-in peptides were more in line with expectation. Additionally the background grey colored peptides showed many false positives before adjustment. After adjustment these false positives were decreased considerably.

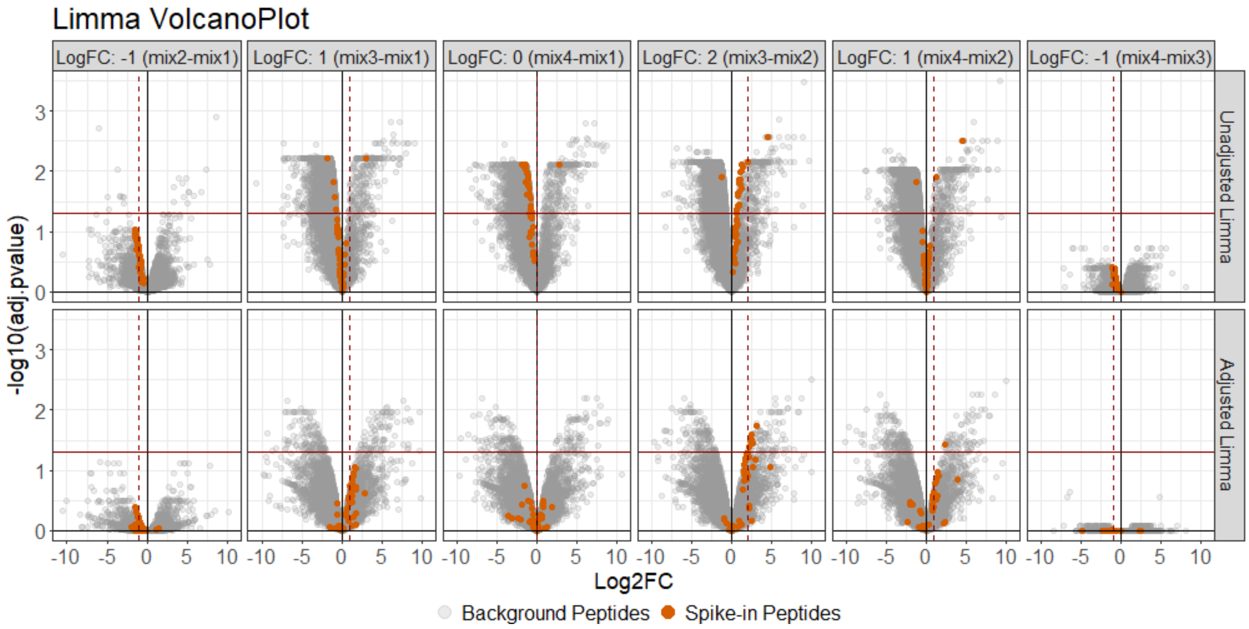

Figure S4: Dataset 3 : SpikeIn benchmark - Ubiquitination - Label-free. When modeling the experiment with the *Limma* method, the spike-in peptides again follow the expected log fold change better after adjusting for changes in protein level. However, while the fold change was more accurate, the majority of spike-in peptides were not detected as differentially abundant. There were more false positive differentially abundant PTM before than after adjustment.

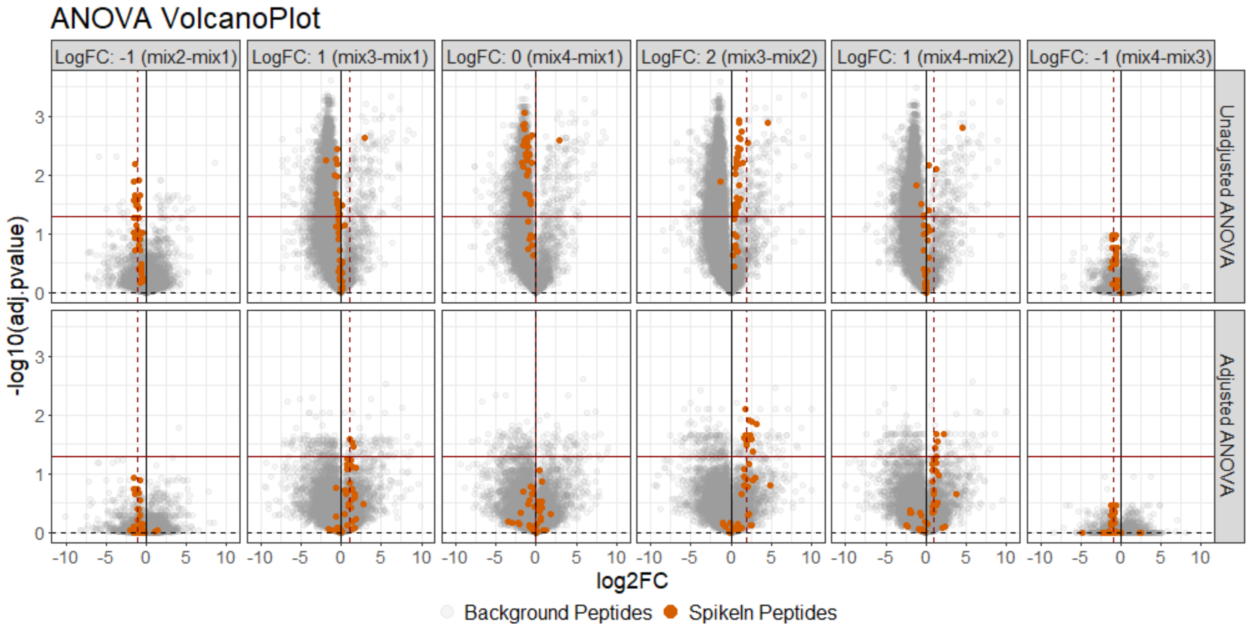

Figure S5: Dataset 3 : SpikeIn benchmark - Ubiquitination - Label-free. Using *ANOVA* the fold change of the spike-in peptides was much closer to expectation after adjusting for global protein abundance. The log FC estimation was the same as *Limma*, however the p-values were different. In this particular case we detect a few more true positives using *ANOVA* compared to *Limma*.

4.3 Dataset 4 : Human - Ubiquitination - 1mix-TMT

The experiment had a simple group comparison design, shown in Table S1.

Condition	BioReplicate	Channel
Dox1hr	Dox1hr_1	127C
Dox2hr	Dox2hr_1	128N
Dox2hr	Dox2hr_2	130C
Dox4hr	Dox4hr_1	128C
Dox4hr	Dox4hr_2	131C
Dox6hr	Dox6hr_1	129N
Dox6hr	Dox6hr_2	131N
NoDox0hr	NoDox0hr_1	126C
NoDox0hr	NoDox0hr_2	129C
NoDox6hr	NoDox6hr_1	127N
NoDox6hr	NoDox6hr_2	130N

Table S1: The experimental design of Dataset 4

The following model was fit separately for ubiquitinated peptides and for unmodified protein

$$Y_{mij} = \mu_i + \epsilon_{mij},\ \sum_{i=1}^{I} \mu_i = 0,\ \epsilon_{mij} \ \sim N(0, \sigma^2)$$

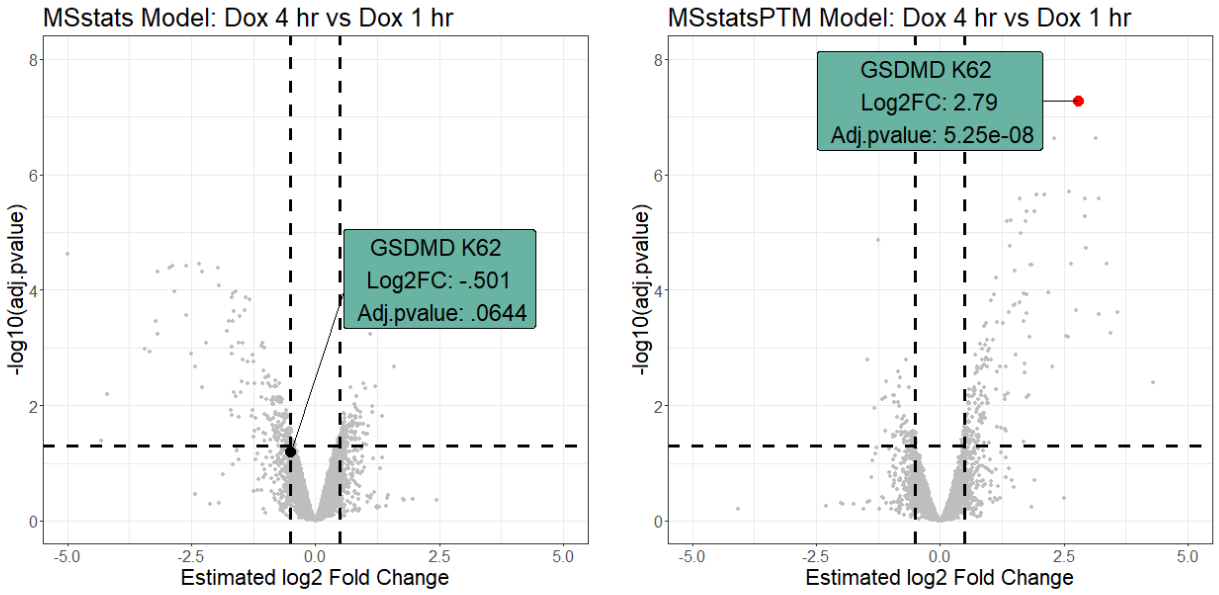

(a) (b)

Figure S6: Dataset 4 : Human - Ubiquitination - 1mix-TMT. Volcano plots of Dox4hr vs Dox1hr both before and after protein adjustment with $MSstatsPTM$. The $GSDMD\_HUMAN|P57764\_K62$ modification is highlighted. (a) Before adjustment the modification had a small fold change and was not detected as differentially abundant. (b) After adjustment the fold change was much larger, and the modification was detected as differentially abundant. In this case $MSstatsPTM$ allowed us to identify a differential modified peptide that could have otherwise been missed.

4.4 Dataset 5 : Mouse - Phosphorylation - 2mix-TMT

The experiment had a group comparison design, and the data were acquired in two mixtures, as shown in Table S2.

	Mixture 1		Mixture 2		Condition
Uninfected	128C		128C	131C	
Early (1 Hour)	126C	129C	126C	129C	WT
Late (3 Hour)	127C	130C	127C	130C	
Uninfected	129N	131C	129N		
Early (1 Hour)	127N	130N	127N	130N	KO
Late (3 Hour)	128N	131N	128N	131N	

Table S2: The experimental design of Dataset 5

The following model was fit separately for phosphorylated peptides and for unmodified protein

$$Y_{mij} = \mu_i + Mixture_m + \epsilon_{mij},\ Mixture_m\ \sim N(0, \sigma_M^2),\ \sum_{i=1}^{I} \mu_i = 0,\ \epsilon_{mij}\ \sim N(0, \sigma^2)$$

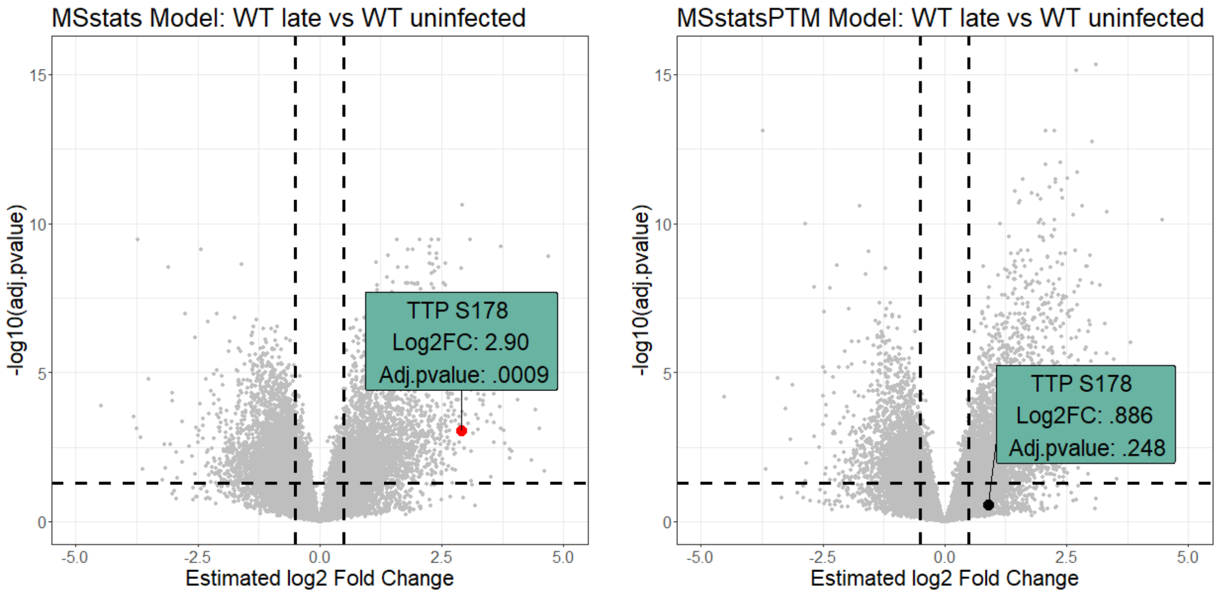

(a) (b)

Figure S7: Dataset 5 : Mouse - Phosphorylation - 2mix-TMT. Volcano plots of WT_Late vs WT_Uninfect both before and after protein adjustment with *MSstatsPTM*. The $TTP\_MOUSE|P22893\_S178$ modification is highlighted. (a) Before adjustment the modification had a large fold change and a small p-value. (b) After adjustment the fold change was much smaller and the modification was not detected as differentially abundant.

References

1. Kirkpatrick, D.S. et al. (2013). "Phosphoproteomic characterization of DNA damage response in melanoma cells following MEK/PI3K dual inhibition". In: *Proceedings of the National Academy of Sciences* 48.110, pp. 19426–19431.

2. Beausoleil, S.A. et al. (2006). "A probability-based approach for high-throughput protein phosphorylation analysis and site localization". In: *Nature Biotechnology* 24, pp. 1285–1292.

3. Bakalarski, C.E. et al. (2008). "The impact of peptide abundance and dynamic range on stable-isotope-based quantitative proteomic analyses". In: *Journal of Proteome Research* 11.7, pp. 4756–4765.
